# Contributions of obesity to kidney health and disease: insights from Mendelian randomization and the human kidney transcriptomics

**DOI:** 10.1093/cvr/cvab357

**Published:** 2021-12-10

**Authors:** Xiaoguang Xu, James M Eales, Xiao Jiang, Eleanor Sanderson, Maciej Drzal, Sushant Saluja, David Scannali, Bryan Williams, Andrew P Morris, Tomasz J Guzik, Fadi J Charchar, Michael V Holmes, Maciej Tomaszewski

**Affiliations:** Division of Cardiovascular Sciences, Faculty of Medicine, Biology and Health, University of Manchester, AV Hill Building, Manchester, M13 9PT, UK; Division of Cardiovascular Sciences, Faculty of Medicine, Biology and Health, University of Manchester, AV Hill Building, Manchester, M13 9PT, UK; Division of Cardiovascular Sciences, Faculty of Medicine, Biology and Health, University of Manchester, AV Hill Building, Manchester, M13 9PT, UK; MRC Integrative Epidemiology Unit, University of Bristol, Oakfield House, Bristol, BS8 2BN, UK; Division of Cardiovascular Sciences, Faculty of Medicine, Biology and Health, University of Manchester, AV Hill Building, Manchester, M13 9PT, UK; Division of Cardiovascular Sciences, Faculty of Medicine, Biology and Health, University of Manchester, AV Hill Building, Manchester, M13 9PT, UK; Division of Cardiovascular Sciences, Faculty of Medicine, Biology and Health, University of Manchester, AV Hill Building, Manchester, M13 9PT, UK; Institute of Cardiovascular Sciences, University College London, Roger Williams Building, London, WC1E 6HX, UK; Centre for Genetics and Genomics Versus Arthritis, Centre for Musculoskeletal Research, Division of Musculoskeletal & Dermatological Sciences, Faculty of Medicine, Biology and Health, University of Manchester, AV Hill Building, Manchester, M13 9PT, UK; BHF Glasgow Cardiovascular Research Centre, Institute of Cardiovascular and Medical Sciences, College of Medical, Veterinary and Life Sciences, University of Glasgow, Glasgow, G12 8TA, UK; Department of Internal and Agricultural Medicine, Jagiellonian University College of Medicine, Skarbowa 1, 31-121 Kraków, Poland; School of Science, Psychology and Sport, Federation University, Ballarat, Victoria, 3353, Australia; Department of Cardiovascular Sciences, University of Leicester, University Road, Leicester, LE1 7RH, UK; Department of Physiology, University of Melbourne, Medical Building 181, Melbourne, Victoria, 3010, Australia; NIHR Oxford Biomedical Research Centre, Oxford University Hospitals NHS Foundation Trust, John Radcliffe Hospital, Oxford, OX4 2PG, UK; Medical Research Council Population Health Research Unit at the University of Oxford, Nuffield Department of Population Health, University of Oxford, Oxford, OX3 7LF, UK; Clinical Trial Service Unit & Epidemiological Studies Unit (CTSU), Nuffield Department of Population Health, University of Oxford, Big Data Institute Building, Roosevelt Drive, Oxford, OX3 7LF, UK; Division of Cardiovascular Sciences, Faculty of Medicine, Biology and Health, University of Manchester, AV Hill Building, Manchester, M13 9PT, UK; Manchester Heart Centre and Manchester Academic Health Science Centre, Manchester University NHS Foundation Trust Manchester, Manchester Royal Infirmary, Oxford Road, Manchester, M13 9WL, UK

**Keywords:** Obesity, Kidney function, Kidney disease, Mendelian randomization, Kidney transcriptome

## Abstract

**Aims:**

Obesity and kidney diseases are common complex disorders with an increasing clinical and economic impact on healthcare around the globe. Our objective was to examine if modifiable anthropometric obesity indices show putatively causal association with kidney health and disease and highlight biological mechanisms of potential relevance to the association between obesity and the kidney.

**Methods and results:**

We performed observational, one-sample, two-sample Mendelian randomization (MR) and multivariable MR studies in ∼300 000 participants of white-British ancestry from UK Biobank and participants of predominantly European ancestry from genome-wide association studies. The MR analyses revealed that increasing values of genetically predicted body mass index and waist circumference were causally associated with biochemical indices of renal function, kidney health index (a composite renal outcome derived from blood biochemistry, urine analysis, and International Classification of Disease-based kidney disease diagnoses), and both acute and chronic kidney diseases of different aetiologies including hypertensive renal disease and diabetic nephropathy. Approximately 13–16% and 21–26% of the potentially causal effect of obesity indices on kidney health were mediated by blood pressure and type 2 diabetes, respectively. A total of 61 pathways mapping primarily onto transcriptional/translational regulation, innate and adaptive immunity, and extracellular matrix and metabolism were associated with obesity measures in gene set enrichment analysis in up to 467 kidney transcriptomes.

**Conclusions:**

Our data show that a putatively causal association of obesity with renal health is largely independent of blood pressure and type 2 diabetes and uncover the signatures of obesity on the transcriptome of human kidney.

## Graphical Abstract

**Graphical Abstract:**
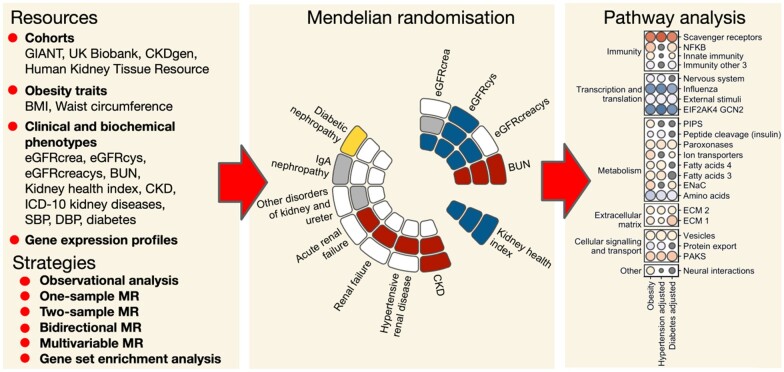

**Time for primary review: 48 days.**

**See the editorial comment for this article ‘Genetically-instrumented public health: facing obesity to prevent chronic kidney disease’, by Ryosuke Fujii and Cristian Pattaro, https://doi.org/10.1093/cvr/cvac168.**

## 1. Introduction

Chronic kidney disease (CKD) affects more than 10% of adults worldwide and is predicted to become a global threat to public health.^1^ Interventions with reliable evidence for effectiveness in preventing CKD and/or slowing the progression of CKD are limited. Amongst the potentially safest to implement, cheapest to introduce, and generally accepted by both patients and clinicians are health behaviours/lifestyle modifications, such as weight loss. Such lifestyle modifications are usually recommended as first-line interventions in primary/secondary prevention of cardiovascular disease (CVD)^2^ and in the management of patients with CKD.^3^ However, it is not clear to what extent modifiable health behaviours are effective in improving clinical indices of kidney health i.e. in slowing the decline in estimated glomerular filtration rate (eGFR). For example, NICE guidelines recommend maintaining a healthy weight since it is a safe ‘healthy life’ strategy rather than due to its nephro-protective effects.^3^

A number of previous investigations reported associations between increasing adiposity/obesity and a decline in kidney function or the increased risk of kidney diseases.^4–10^ However, it is not clear to what extent these associations reflect causality, i.e. a cause and effect relationship between obesity and renal health/disease. This is of considerable relevance to clinical management given that only health behaviours with evidence of causal effects on disease (and/or its defining traits) are likely to succeed in effective prevention and treatment.^11^ In contrast, interventions targeting factors external to the disease-related causal pathways often fail to deliver the expected clinical outcomes.^12^

Mendelian randomization (MR) has emerged as a genetic epidemiological approach that enables causal inference (analogous to a natural randomized controlled trial—RCT) and a powerful alternative and/or complement to the conventional RCT.^12^ A recent review by Friedman *et al.*^13^ summarized the evidence from RCTs assessing the effect of weight loss interventions on micro-/macro-albuminuria, changes in eGFR and/or serum creatinine. Some of these studies demonstrated an improvement in renal outcomes with weight reduction^14–19^; while others demonstrated no significant effect.^20,21^ Conducting RCTs is generally very expensive, logistically challenging, risky (as the intervention may not demonstrate clinical efficacy), time-consuming and under certain circumstances—may not be free of clinical hazards.^22^ In contrast, MR does not require the resources or time consumed by RCT, is safe and generally robust.^23,24^ It relies on randomly assigned (at meiosis) genetic variants as proxies (or instruments) for an exposure (e.g. a modifiable lifestyle factor) to examine its putative causal effect on a clinical outcome. Compared to findings from observational studies, those from MR are less susceptible to bias from many sources of unmeasured confounding and reverse causality.^25,26^ MR has been increasingly applied to exposures/outcomes of direct relevance to clinical nephrology. For example, previous MR-based investigations uncovered causality signals to or from renal phenotypes, e.g. an effect of eGFR on diastolic blood pressure (DBP) and risk of nephrolithiasis,^27^ the causal connection between higher blood pressure (BP) and the risk of microalbuminuria,^28^ as well as hypertensive renal disease and CKD.^29^ Most recently, based on studies embedded largely in one-sample MR, Censin *et al.*^30^ and Zhu *et al.*^31^ showed that increasing values of body mass index (BMI)/waist–hip ratio were associated with increased risk of renal failure/CKD.

Herein, using both one-sample and complementary two-sample MR analyses, we have shown that obesity-related traits show potentially causal association with many different dimensions of kidney heath and disease—from physiological measures of kidney function to kidney diseases of several aetiologies. Furthermore, through analysis of the largest collection of renal gene expression profiles characterized by RNA-sequencing,^27,29,33–36^ we characterized how increasing values of obesity measures translate into changes in molecular pathways and biological themes operating in the kidney. Finally, through exploitation of information derived from kidney transcriptomes, we provide important biological context to statistical findings on hypertension and diabetes as potential mediators of causal associations between obesity and renal health/disease contemplated in this and other publications.^13,31^

## 2. Condensed methods

**Figure 1:**
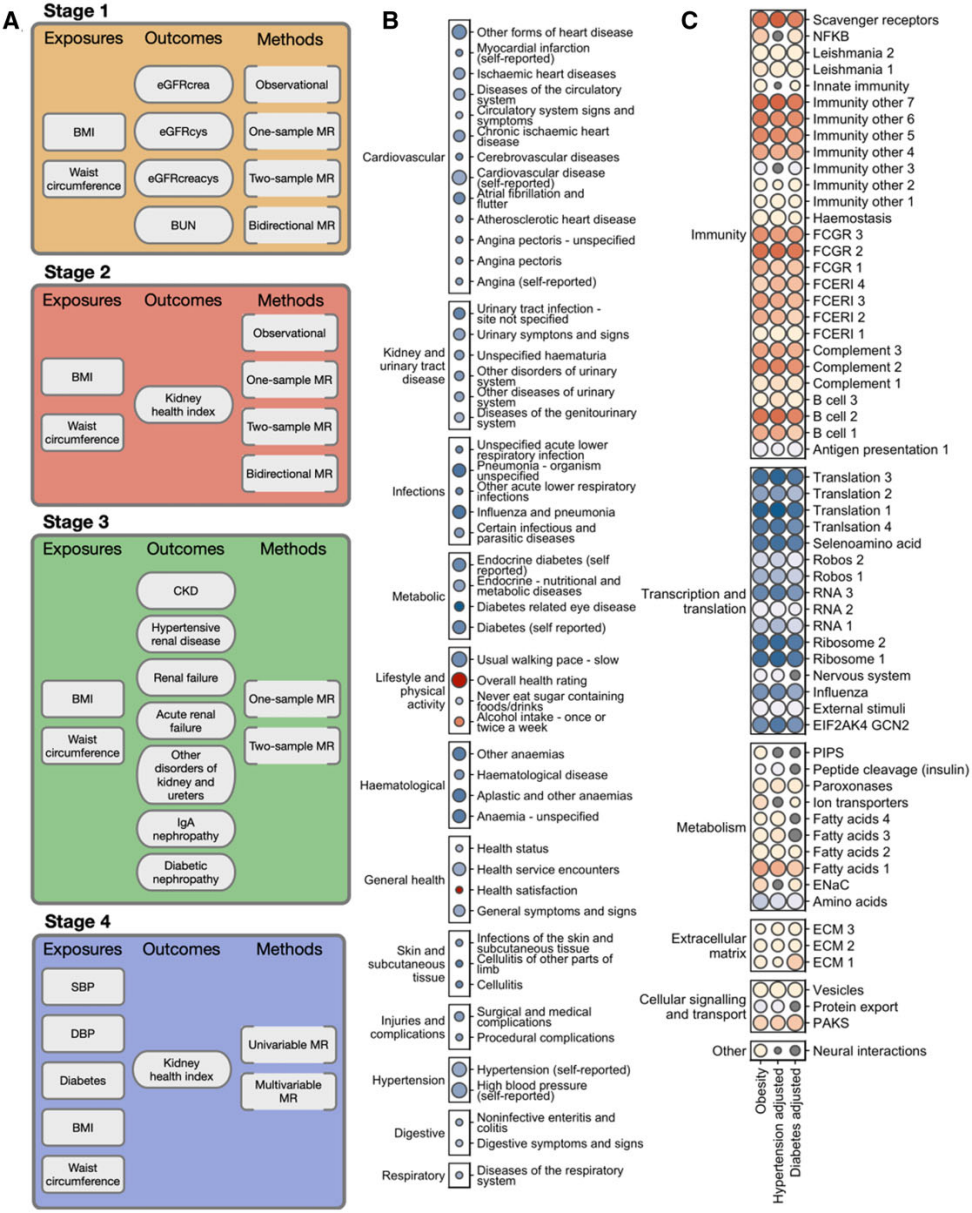
Overview of the strategy and the outputs of the study. (*A*) Conceptual overview of the study in four stages of MR studies. Stage 1—obesity traits and biochemical measures of kidney function, Stage 2—obesity traits and kidney health index, Stage 3—obesity traits and kidney disease, Stage 4—hypertension and diabetes as potential mediators of association between obesity traits and kidney health index. eGFRcrea, GFR estimated by creatinine; eGFRcys, GFR estimated by cystatin C; eGFRcreacys, GFR estimated by creatinine and cystatin C; BUN, blood urea nitrogen; SBP, systolic blood pressure; DBP, diastolic blood pressure; kidney health index, a composite renal phenotype integrating all available serum measures of kidney function (eGFRcrea, eGFRcys, eGFRcrea, and BUN), a uACR and the ICD-derived information on the history of kidney disease from hospital episode statistics. (*B*) Summary of the 50 most highly correlated phenotypes with kidney health index. Strength of statistical significance is shown by the size of the coloured circle, direction, and magnitude of association is shown by intensity of either blue (negative association) or red (positive association). All phenotypes are grouped and labelled by clinical category. (*C*) Summary of GSEA on canonical pathways for obesity traits alone and after adjustment for hypertension and diabetes. Strength of statistical significance is shown by the size of the coloured circle, direction, and magnitude of association is shown by intensity of either blue (negative association) or red (positive association), non-significant results after adjustment are coloured grey. All pathways are grouped and labelled by their biological theme. NFKB, ST Ga13 pathway; FCGR1, Reactome fcgamma receptor FCGR dependent phagocytosis; FCGR2, Reactome FCGR activation; FCGR3, Reactome FCGR3A-mediated IL10 synthesis; FCERI1, Reactome Fc epsilon receptor FCERI signalling; FCERI2, Reactome FCERI mediated Ca+2 mobilization; FCERI3, Reactome FCERI mediated MAPK activation; FCERI4, Reactome FCERI mediated NF-kB activation; Robos1, Reactome regulation of expression of SLITs and ROBOs; Robos2, Reactome signaling by ROBO receptors; RNA1, Reactome RRNA processing; RNA2, Reactome metabolism of RNA; RNA3, Reactome Nonsense-Mediated Decay; PIPS, Reactome synthesis of PIPS at the Golgi membrane; ENaC, Epithelial sodium channel; ECM1, Reactome MET activates PTK2 signaling; ECM2, Reactome MET promotes cell motility; ECM3, Reactome signaling by MET; PAKS, Reactome RHO GTPases activate PAKs.

An overview of our strategy and undertaken analyses are shown in *Figure 1A* and Supplementary material online, *Table S1*. Full details of methods are provided in Supplementary material online. If not specified otherwise, all the statistical analyses were performed using R (version 3.6.2).

### 2.1 UK Biobank—key phenotypes

UK Biobank is a population-based resource on 487 395 individuals with a wide range of clinical data linked to genetic information. From this dataset, we extracted information on the two most common anthropometric measures of obesity [BMI and waist circumference (WC)] and four measures of kidney function (*Figure 1A*) in 337 422 individuals of European ancestry. In brief, we used information on blood urea nitrogen (BUN) and three measures of eGFR derived from: serum creatinine (eGFRcrea), cystatin C (eGFRcys), and both creatinine and cystatin C (eGFRcreacys). The number of individuals with informative values of eGFRcrea, eGFRcys, eGFRcreacys and BUN was 304 800, 303 373, 317 425, and 314 731, respectively. We further generated the kidney health index—a novel composite renal phenotype integrating all available serum measures of kidney function (eGFRcrea, eGFRcys, eGFRcrea, and BUN), a urinary biomarker of kidney damage [albumin-to-creatinine ratio (uACR)] and the International Classification of Disease (ICD)-derived information on the history of kidney disease from Hospital Episodes Statistics (Supplementary material online, *Figure S1*).^37^ A total of 217 289 individuals satisfied the criteria of optimal kidney health index and were defined as having optimal kidney health. The remaining 84 657 individuals did not meet at least one of the criteria of the kidney health index and were defined as not having optimal kidney health.

### 2.2 Observational analysis in UK Biobank

To characterize the observational association between directly measured BMI/WC and quantitative serum biomarkers of kidney function (eGFRcrea, eGFRcys, eGFRcreacys, and BUN), we used linear regression. We applied logistic regression to examine the association between BMI/WC and the binary kidney health index. All these regression models were adjusted for age, age^2^, sex, assessment centre, and Townsend Deprivation Index.

We selected all binary traits with a number of cases >100 from self-reported data and ICD10-derived diagnoses available in UK Biobank (Supplementary material online, *Table S2*). These traits were further grouped into 22 clinical categories (Supplementary material online, *Table S2*). We explored the association between kidney health index (as an independent variable) and 403 binary traits (as a dependent variable) through logistic regression (with age, age^2^, sex, assessment centre, and Townsend Deprivation Index as covariates). We calculated a correction for multiple testing using the false discovery rate (FDR)—findings with FDR < 0.05 were considered statistically significant.

### 2.3 Selection of genetic instruments

To build genetic instruments and generate genetic scores for BMI and WC, we followed the strategy from a previous study.^38^ In brief, we used 72 and 43 instruments from SNPs associated with BMI and WC, respectively, in genome-wide association study (GWAS) conducted in 339 224 and 224 459 individuals of European ancestry independent of UK Biobank by GIANT Consortium^39,40^ and validated in previous MR analysis.^38^ To minimize the risk that potential causality signals from BMI/WC to kidney phenotypes may reflect an effect on metabolism of creatinine/cystatin C/BUN rather than renal function, we further re-evaluated the associations by excluding SNPs within a distance of 500 kb of genes recognized for their roles in the metabolism of these blood biomarkers (Supplementary material online, *Table S3*). We further re-examined our MR models excluding instruments showing residual associations with smoking and educational attainment in PheLiGe^41^ (Supplementary material online, *Table S4*). To examine if the effect of obesity on kidney-related traits was mediated by BP or diabetes, we further derived instruments/genetic scores for systolic blood pressure (SBP), DBP, and type 2 diabetes (T2D). In the absence of summary statistics for BMI-unadjusted BP from previously published GWAS, we applied a block jack-knife weighting approach^42^ to perform GWAS on SBP/DBP in UK Biobank. For T2D, we used the summary statistics from largest available GWAS conducted in individuals of European ancestry^43^ that were not BMI-adjusted and independent of UK Biobank.

### 2.4 One-sample MR in UK Biobank

We applied one-sample MR [two-stage least square approach (2SLS)^44^] with externally derived genetic scores of BMI, WC, SBP, DBP, and T2D as an instrument, measured BMI, WC, SBP, DBP, and T2D as an exposure, kidney function (i.e. eGFRcrea, eGFRcys, eGFRcreacys, and BUN), kidney health index, ICD-informed kidney diagnoses (CKD, hypertensive renal disease, renal failure, and acute renal failure or other disorders of kidney and ureters, where appropriate) as an outcome (*Figure 1A*). Age, age^2^, sex, genotyping array, and first 10 genetic principal components were used as covariates. We calculated a correction for multiple testing at each experiment level using FDR—findings with FDR < 0.05 were considered statistically significant.

### 2.5 Two-sample MR

We conducted two-sample MR using four different models [inverse variance weighted (IVW) regression, weighted median, RadialMR, and MRPRESSO]^45–47^ to: (i) replicate the estimated causal effects of both obesity indices (as exposures) on biochemical parameters of kidney function; (ii) further investigate potentially causal effects of obesity on kidney diseases (CKD, IgA nephropathy, and diabetic nephropathy) (*Figure 1A*); (iii) validate causal effects of BMI and WC on kidney health index in a sensitivity analysis, and (iv) explore the existence of reverse causality (i.e. causal effects from eGFRcys, BUN, and kidney health index on obesity indices) (*Figure 1A*). Weighted median, RadialMR, and MRPRESSO were chosen to minimize any potential bias arising from horizontal pleiotropy.^48^ To further quantify the magnitude of horizontal pleiotropy, we employed MR-Egger intercept test.^45^ Summary statistics for exposures and outcomes, and the selection of genetic instruments were derived from UK Biobank, CKDGen Consortium,^49^ and other previous GWAS.^39,40,50,51^ To correct for multiple testing, we calculated FDR at the experiment-wide level. Causal effect estimates from at least three of the four MR methods significant after the correction for multiple testing (FDR < 0.05) and no evidence of horizontal pleiotropy (FDR > 0.05) were set as a criterion of evidence for causality. RadialMR and MRPRESSO were performed using R packages RadialMR (version 0.4) and MRPRESSO (version 1.0), respectively. IVW, weighted median and MR-Egger intercept test were implemented in the R package MendelianRandomization (version 0.4.2).

### 2.6 One-sample multivariable MR analysis

We conducted multivariable MR (MVMR) with BMI, WC, SBP, DBP, and T2D as exposures and kidney health index as the outcome using a 2SLS approach.^52^ In brief, MVMR estimations measure the direct effect of each exposure on the outcome adjusted for the other exposures included in the model. We examined several separate combinations of BMI/WC with SBP/DBP/T2D, with the genetic scores associated with all exposures included in the derivation of predicted estimates. The proportion of effect mediated by mediators (i.e. T2D only, BP only or T2D and BP together) was derived by dividing the indirect effect by the total effect, with standard error estimated using bootstrapping.^53^ Age, age^2^, sex, genotyping array, and first 10 genetic principal components were used as covariates. We calculated a correction for multiple testing using FDR—findings with FDR < 0.05 were considered statistically significant.

### 2.7 Kidney transcriptome profiling and pathway analyses

We collected demographic and clinical information data on up to 467 human kidney tissue samples (Supplementary material online, *Table S5*) drawn from five studies of the Human Kidney Tissue Resource.^29,32–36^ The tissue samples were taken either from the unaffected pole of the kidneys surgically removed due to cancer or from pre-implementation biopsies conducted prior to kidney transplantation.^35^ Gene expression was quantified in units of transcripts per million by kallisto^54^ from poly-A selected Illumina libraries (mean: 32 million paired reads per sample). Our quality control process selected 22 127 renal genes for further analysis—their expression values were log-transformed, normalized by the robust quantile method and standardized by the rank-based inverse normal transformation as previously reported.^35^ BMI was calculated based on weight and height as reported before.^35^ WC was measured using a measuring tape placed around the trunk at the midline level. Hypertension was defined as BP values ≥140/90 mmHg on at least two separate occasions^33,35^ and/or being on pharmacological antihypertensive treatment or collected from hospital documentation by the recruitment team.^35^ Diabetes was defined as either a self-reported history of diabetes and/or being on hypoglycaemic medications or was reported in hospital documentation at the time of recruitment.^32,35^ We applied Gene set enrichment analysis (GSEA, www.bioconductor.org/packages/release/bioc/html/fgsea.html) for the ‘canonical pathways’ collection from the molecular signatures database (v7.2, www.gsea-msigdb.org) to identify pathways associated with either BMI or WC. The analysis was conducted separately for each of the phenotypes after adjustment for age, sex, study, three genetic principal components,^29^ sequencing batch and either 30 (BMI) or 33 (WC) surrogate variables.^35,55^ Pathways showing significant (adjusted level of statistical significance after correction for multiple testing) association with BMI and WC were combined and mapped onto biological themes based on the pathway identity and leading edge genes in line with previously reported strategy.^56^ We then added either hypertension or diabetes as individual covariates to the GSEA models and examined the effect of this adjustment on the number and identity of the pathways in the original output. The original pathways retaining their directionally consistent (based on normalized enrichment statistic) significant association with BMI or WC after adjusting for hypertension or diabetes were deemed as independent of hypertension and diabetes (respectively) while those whose association was no longer statistically significant after the adjustment were interpreted as being mediated specifically either by hypertension or diabetes.

### 2.8 Ethical compliance

The studies adhered to the Declaration of Helsinki and were approved/ratified by the Bioethics Committee of the Medical University of Silesia (Katowice, Poland), the Bioethics Committee of Karol Marcinkowski Medical University (Poznan, Poland), the Ethics Committee of the University of Leicester (Leicester, UK), the University of Manchester Research Ethics Committee (Manchester, UK), and the National Research Ethics Service Committee North West (Manchester, UK). Informed, written consents were obtained from all individuals recruited (for the deceased donors, the consent was obtained in line with the local governance; for example, from the family members).

## 3. Results

### 3.1 Measures of general and abdominal obesity are causally associated with biochemical parameters of kidney function

We first used data from up to 337 422 UK Biobank individuals of European ancestry (Supplementary material online, *Table S6*) to examine how BMI and WC relate to serum parameters of kidney function (eGFRcrea, eGFRcys, eGFRcreacys, and BUN) (*Figure 1A*). Both directly measured obesity indices showed significant associations with each renal phenotype in the observational analysis, even after a correction for multiple testing (Supplementary material online, *Figures S2* and *S3*). Using genetically predicted information for BMI and WC in one-sample MR, we detected causal relationships of both obesity measures with three indices of kidney function (eGFRcys, eGFRcreacys, and BUN) consistent with increasing obesity translating into poorer kidney function (Supplementary material online, *Figures S2* and *S3*). For example, each 5 kg/m^2^ genetically predicted higher BMI was causally related to −0.041 [95% confidence interval (CI): −0.047 to −0.037; *P* = 5.96 × 10^−59^] and −0.02 (95% CI: −0.023 to −0.015; *P* = 1.78 × 10^−21^) unit change in log-transformed eGFRcys and eGFRcreacys and 0.022 (95% CI: 0.015–0.028; *P* = 2.01 × 10^−10^) unit change in log-transformed BUN. We then replicated a directionally consistent effect of BMI and WC on eGFRcys and BUN using CKDGen Consortium summary statistics (Supplementary material online, *Table S7* and *Figures S2* and *S4*). Further sensitivity analyses confirmed that association estimates between obesity measures and measures of kidney function remained largely unaffected by inclusion/exclusion of genetic variants mapping onto genes related to metabolism of creatinine/cystatin C/BUN (Supplementary material online, *Table S3*) and that there is no evidence for bidirectional causality between obesity measures and kidney function (Supplementary material online, *Table S8*). Collectively, these results show a consistent and potentially causal association between higher BMI and WC and kidney function across different MR models and in independent datasets.

### 3.2 Obesity measures show a causal inverse association with kidney health index

Mindful of the potential limitation of using circulating concentrations of specific biomarkers as a marker of kidney function in MR,^57^ we generated a ‘kidney health index’ as a composite binary phenotype derived from the blood and urine biochemistry combined with available clinical information in UK Biobank^37^ (*Figure 1A* and Supplementary material online, *Figure S1* and *Methods*). The index is using ‘health’ rather than ‘disease’ as a reference. To this end and as expected, those with optimal kidney health index (corresponding to ‘healthy’ kidneys) were associated with favourable renal profile across different serum biomarkers (eGFRcrea, eGFRcys, eGFRcreacys, and BUN) and uACR when compared to the remaining individuals (Supplementary material online, *Table S9*). In observational analysis, both BMI and WC were inversely related to the kidney health index (Supplementary material online, *Table S10* and *Figure S2*). Further analysis of 403 binary traits in UK Biobank revealed strong associations between kidney health index and phenotypes expected to correlate with measures of kidney health and disease (e.g. kidney and urinary tract-related traits, CVD, hypertension, diabetes, and metabolic diseases) (*Figure 1B* and Supplementary material online, *Table S11*). Indeed, the directions of these associations were consistent with reduced odds of these disorders in those with optimal health index category. We then combined GIANT consortium-derived BMI and WC genetic scores and the kidney health index as an outcome in one-sample MR. This analysis revealed that each 5 kg/m^2^ genetically predicted higher BMI and 10 cm genetically predicted higher WC were causally associated with decreased odds of kidney health index by 15 and 14% [odds ratio (OR) = 0.85; 95% CI: 0.79–0.91; *P* = 9.18 × 10^−6^ for BMI and OR = 0.86; 95% CI: 0.81–0.92; *P* = 2.12 × 10^−5^ for WC, respectively] (Supplementary material online, *Table S10*). We then conducted several sensitivity analyses. Mindful of the potential limitations of genetic risk scores constructed based on previous GWAS of obesity traits^30,31,38^ and biomarker metabolism related instruments,^57^ we examined the effect of BMI and WC on kidney health index after exclusion of SNPs showing associations with smoking and education in PheLiGe^41^ or those related directly to genes metabolically or mechanistically involved in BUN, creatinine, and cystatin C metabolism.^57^ These sensitivity analyses confirmed statistically significant putatively causal associations of both anthropometric measures of obesity with kidney health index (Supplementary material online, *Tables S12* and *S13*). Finally, we employed two-sample MR tests with GIANT-derived genetic instruments for BMI and WC (as exposures) and the summary statistics from our *de novo* GWAS of kidney health index (as an outcome) in UK Biobank. These analyses confirmed the findings from one-sample MR (*P*_BMI_IVW_ = 2.27 × 10^−5^ and *P*_WC_IVW_ = 1.54 × 10^−4^) (Supplementary material online, *Table S10* and *Figure S2*) and showed no evidence for bidirectional causality between obesity measures and kidney health index (Supplementary material online, *Table S14*). Taken together, these results suggest that obesity measures may have a potentially causal inverse effect on the kidney health index.

### 3.3 BMI and WC are causally related to increased risk of different kidney diseases

We then sought to investigate if BMI/WC were causally related to clinically confirmed kidney outcomes using both one-sample and two-sample MR studies (*Figure 1A*). Using GIANT Consortium-derived genetic scores for BMI and WC and ICD10-derived diagnoses from Hospital Episodes Statistics, we first conducted a series of one-sample MR in UK Biobank. We detected a causal relationship between obesity measures and four out of five kidney diagnoses—each 5 kg/m^2^ genetically predicted higher BMI and/or each 10 cm genetically predicted higher WC increased the risk of hypertensive renal disease (OR = 1.89; 95% CI: 1.15–3.09; *P* = 1.15 × 10^−2^ for BMI and OR = 1.94; 95% CI: 1.22–3.08; *P* = 5.03 × 10^−3^ for WC), renal failure (OR = 1.57; 95% CI: 1.27–1.94; *P* = 2.60 × 10^−5^ for BMI and OR = 1.59; 95% CI: 1.31–1.94; *P* = 4.16 × 10^−6^ for WC), and CKD (OR = 1.56; 95% CI: 1.17–2.08; *P* = 2.44 × 10^−3^ for BMI and OR = 1.58; 95% CI: 1.21–2.07; *P* = 9.49 × 10^−4^ for WC) (Supplementary material online, *Table S15* and *Figure S2*). We then used two-sample MR using GWAS summary statistics of three kidney outcomes (CKD, IgA nephropathy, and diabetic nephropathy) from populations independent to UK Biobank. These analyses replicated a causal effect of obesity on CKD (OR_ivw_ = 1.18; 95% CI: 1.05–1.33; *P* = 6.07 × 10^−3^ for BMI and OR_ivw_ = 1.13; 95% CI: 1.002–1.284; *P* = 4.66 × 10^−2^ for WC) and uncovered a causal relationship between BMI and diabetic nephropathy (OR_ivw_ = 2.03; 95% CI: 1.49–2.77; *P* = 6.80 × 10^−6^) (Supplementary material online, *Table S16* and *Figure S2*). Collectively, these data show that obesity increases the risk of acute and CKD of several aetiologies.

### 3.4 The causal association between obesity measures on kidney health index is largely independent of blood pressure and T2D

Elevated BP and diabetes have been proposed as the most likely biological mediators of the association between obesity and the risk of kidney disease.^58,59^ Therefore, we examined whether SBP, DBP, and T2D are causally related to the kidney health index in UK Biobank (*Figure 1A* and Supplementary material online, *Tables S17* and *S18*). SBP, DBP, and T2D showed potentially causal association with kidney health index in the expected (inverse) direction (*P*_SBP_ = 6.26 × 10^−6^, *P*_DBP_ = 1.69 × 10^−2^, and *P*_T2D_ = 6.13 × 10^−11^, respectively) (Supplementary material online, *Tables S17* and *S18*). Using one-sample multivariable MR,^52^ we then explored the extent to which the detected signals of potential causality between BMI/WC and kidney health index were mediated by BP and T2D. These analyses revealed that 13–16% of the potentially causal effect of obesity parameters on kidney health was mediated by BP and 21–26% by T2D (Supplementary material online, *Table S19*). SBP or DBP and T2D jointly accounted for 26–34% proportion of causal association of BMI/WC and kidney health index (Supplementary material online, *Tables S17–S20*). Collectively, these data suggest that potentially causal negative effect of obesity on kidney health is only partly mediated by BP and T2D.

### 3.5 Kidney pathways and biological themes associated with BMI and WC—insights from GSEA of kidney transcriptomes

To gain insights into potential biological mechanisms underpinning the associations between obesity and the kidney, we have conducted BMI/WC-based GSEA of up to 467 renal transcriptomes characterized by RNA-sequencing, as reported before.^29,32,34^ We identified a total of 61 pathways mapping predominantly onto transcriptional and translational regulation, innate and adaptive immunity, extracellular matrix remodelling, and different dimensions of human metabolism (Supplementary material online, *Table S21* and *Figure 1C*). The pattern of renal gene expression emerging from BMI/WC-based GSEA was consistent with up-regulation of B cells, complement system and nuclear factor κB response, and increased pro-fibrotic signalling (i.e. through components of extracellular matrix) accompanied by alterations in pathways involved in sodium (i.e. epithelial sodium channels—ENaCs), creatinine, and urate homeostasis (e.g. organic cation/anion zwitterion transporters) as well as signalling cascades operating at the intersection of metabolism and structural kidney injury (i.e. fatty acid uptake and accumulation regulators) (Supplementary material online, *Table S21* and *Figure 1C*). Taking advantage of the availability of clinical information matching the kidney transcriptomes, we adjusted the baseline GSEA analysis for either hypertension or diabetes and compared the extent to which each of these adjustments will attenuate the associations with BMI and WC. These adjusted analyses revealed that 54 (89%) pathways showing association with obesity measures in the unadjusted analysis retained their statistical significance after adjustment for either hypertension or diabetes (Supplementary material online, *Table S21*).

## 4. Discussion

Our results demonstrate putatively causal association of obesity with kidney health and disease, irrespective of the type of MR experiment (one-sample or two-sample) or MR modalities applied and across different biochemical parameters of kidney function as well as a wide spectrum of kidney health/disease. Our analyses also suggest that the potentially causal relationship between indices of obesity and kidney health is largely independent of BP and T2D. Finally, through analysis of up to 467 renal tissue samples with matching clinical data, we uncover identities of 61 biological pathways—signatures of obesity on the transcriptome of the human kidney.

The association between BMI and/or WC and different measures of kidney health and disease have been reported before in observational (both cross-sectional and longitudinal) studies—increasing obesity correlates with increased incidence of CKD,^4–7^ end-stage renal disease,^8^ and a drop in eGFR calculated based on serum levels of creatinine^9^ or cystatin C.^5^ The results from our initial observational analysis of >300 000 individuals are fully consistent with these findings. However, due to inherent limitations of observational analyses (including confounding and reverse causality)^26^ these data cannot provide insights into causal contributions of obesity indices to eGFR and as such are not sufficient to inform i.e. effective preventive strategies. To this end, the MR-derived findings showing putatively causal associations of higher BMI and WC with a decline in kidney function are an important piece of evidence in favour of a potential utility of preventative interventions targeting BMI and/or WC to improve eGFR in individuals largely unaffected by CKD (in whom the relevance of obesity to kidney function is possibly most apparent).^5,10^ Both routine engagement in moderate-intensity regular physical exercise and different dietary interventions have been shown to achieve clinically relevant changes in both WC and BMI.^60^ In this context, our data suggest that non-pharmacological strategies promoting weight loss may have potentially nephro-protective effects.

We examined SBP, DBP, and T2D, as potential mediators of the effect of obesity on kidney health given the established role of both diabetes and hypertension in the development of CKD.^61,62^ Indeed, both diabetes and hypertension showed strong association with the kidney health index in our observational analysis across a wide range of clinical phenotypes in UK Biobank. The potentially causal association between higher BP/T2D and lower odds of healthy kidneys revealed by our MR analyses lends further support to the notion that poorly controlled hypertension^63^ and derangement in glycaemic control^64^ are both detrimental to the kidney function and structure. Our data suggest that only a small to moderate proportion of the effect of obesity on kidney health is accounted for by blood pressure and T2D. The recent investigation by Zhu *et al.*^31^ also showed that BP and T2D could not fully explain the effect of obesity measures on kidney disease but the estimates for mediating effect of BP and T2D diabetes on CKD were more substantial, possibly due to the differences in statistical approach and definition of the outcomes between the studies. Irrespective of the strategy applied to quantification of the mediating effect of hypertension/T2D between Zhu *et al.*^31^ and our study, even jointly high BP and diabetes are unlikely to fully explain the detrimental effect of obesity on kidney health as well as the risk of kidney diseases. To this end, several other BP/glycaemia-independent mechanisms have been contemplated as potential contributors to nephron injury in obesity.^13,64,65^ In search of biological pathways, themes and putative mechanisms underpinning the connection between human obesity and the kidney, we explored one of the largest collections of kidney transcriptomes with matching clinical information^27,29,32–36^ and uncovered enrichment of BMI/WC for innate and adaptive immunity, extracellular matrix remodelling and different dimensions of human metabolism with relatively modest presence of pathways with strong prior relevance to BP regulation or diabetes. Amongst those few known for their role in hypertension was a pathway driven by ENaC genes (i.e. SCNN1B and SCNN1G)—molecular targets for a blood pressure lowering agent (amiloride).^66^ Up-regulation on these genes on the surface of tubular epithelium is indeed a feature of high BP accompanied by obesity.^67^ In a similar vein, few pathways associated with BMI and/or WC were led by genes known for their role in insulin resistance and diabetes (i.e. fatty acid metabolism genes).^68^ Reassuringly, while in numerical minority to other gene sets from GSEA outputs, the pathways with prior physiological connection to hypertension and diabetes emerged as the strongest putative molecular drivers of the mediating effect of T2D diabetes and hypertension in our statistical analyses. Collectively, these data illustrate renal pathways and themes associated with obesity and suggest that a majority of them do not map onto the most obvious molecular cascades of BP regulation and/or glucose and insulin metabolism.

We appreciate that while we optimized our statistical pipeline to ensure the highest possible robustness of our genetic instruments [i.e. independently and strongly associated with obesity (linkage disequilibrium *R*^2^ < 0.01 and *P* < 5 × 10^−8^) validated in the previous MR analysis^38^] and detected no strong evidence of horizontal pleiotropy or ‘bidirectional causality’^69^ some of the instruments may show residual association e.g. with smoking, as reported before.^30,31^ However, our further sensitivity analyses, e.g. those based on exclusion of such instruments from genetic scores confirmed the validity of our findings. We should also highlight interpretational limitations of potential causality signals to eGFR measured using specific biochemical biomarkers.^57^ For example, causal associations between obesity indices and eGFR have been postulated to reflect the effects of specific exposures on the metabolism of specific biomarkers used to estimate kidney function (such as creatinine, cystatin C, or BUN) rather than glomerular filtration *per se.*^57,70^ However, a number of mitigation strategies (e.g. using a range of different indices of kidney function generated using different serum biomarkers as outcomes, eliminating variants mapped to genes metabolically or mechanistically related to the biomarkers from the genetic instruments in sensitivity analyses) have been applied to ensure that the findings from our MR studies were robust to these limitations. Finally, we carried out MR studies using a composite kidney phenotype that integrates information not only from all available blood biochemistry but also—urine analysis and clinical records. The diversity of sources used to generate kidney health index increased immunity of this composite phenotype to bias arising from solitary sources of biochemical and clinical information and possibly make it more reflective of genuine kidney health than individual clinical and biochemical indices. Indeed, our observational analysis on correlating kidney health index with over 400 traits in UK Biobank suggests that it may capture information from a wide range of renal phenotypes. Nevertheless, future studies will be necessary to examine further its temporal associations with relevant clinical outcomes i.e. using time-to-event strategies to define its potential diagnostic and predictive utility.

In summary, our data from a set of statistically robust models embedded in the principles of causal inferences highlighted potentially casual inverse associations between two most common clinical indices of obesity and a range of kidney health and disease-related phenotypes. These findings suggest that obesity has a putatively negative effect on kidney health and increases the risk of different kidney disorders across a spectrum of different aetiologies. Consequently, interventions targeting obesity have a potential to improve kidney health at the population level. We anticipate that our findings will help to stimulate further research and drive the development of public health policies to improve kidney health and prevent kidney disease through encouraging weight loss.

## Supplementary Material

cvab357_Supplementary_Data

## Data Availability

The data underlying this article will be shared on reasonable request to the corresponding author. Translational perspective These findings indicate that obesity is causally linked to indices of renal health and the risk of different kidney diseases. This evidence substantiates the value of weight loss as a strategy of preventing and/or counteracting a decline in kidney health as well as decreasing the risk of renal disease.
