# Supplementary material for: Contributions of obesity to kidney health and disease: insights from Mendelian randomization and the human kidney transcriptomics
Source: Cardiovasc Res. 2021 Dec 10;118(15):3151–61. doi: 10.1093/cvr/cvab357 (PMC9732514; doi:10.1093/cvr/cvab357)
Supplement: cvab357_Supplementary_Data [file cvab357_supplementary_data.zip › Supplementary_appendix_submission_CVR_clean.docx]

**Contributions of obesity to kidney health and disease – insights from Mendelian randomisation and the human kidney transcriptomics**

**Supplement**

Xiaoguang Xu, PhD^1^, James M. Eales, PhD^1^, Xiao Jiang, PhD^1^, Eleanor Sanderson, PhD^2^, Maciej Drzal MSc^1^, Sushant Saluja MBBCh^1^, David Scannali, BSc^1^, Bryan Williams, MD^3^,

Andrew P. Morris, PhD^4^, Tomasz J. Guzik, MD^5,6^, Fadi J. Charchar, PhD^7,8,9^,

Michael V. Holmes, PhD^10,11,12^, Maciej Tomaszewski, MD^1,13^

^1^Division of Cardiovascular Sciences, Faculty of Medicine, Biology and Health, University of Manchester, Manchester, UK

^2^MRC Integrative Epidemiology Unit, University of Bristol, Bristol, UK

^3^Institute of Cardiovascular Sciences, University College London, London, UK

^4^Centre for Genetics and Genomics Versus Arthritis, Centre for Musculoskeletal Research, Division of Musculoskeletal & Dermatological Sciences, Faculty of Medicine, Biology and Health, University of Manchester, Manchester, UK

^5^Institute of Cardiovascular and Medical Sciences, College of Medical, Veterinary and Life Sciences, University of Glasgow, Glasgow, UK

^6^Department of Internal and Agricultural Medicine, Jagiellonian University College of Medicine, Kraków, Poland

^7^School of Health and Life Sciences, Federation University Australia, Ballarat, Victoria, Australia

^8^Department of Cardiovascular Sciences, University of Leicester, Leicester, UK

^9^Department of Physiology, University of Melbourne, Melbourne, Victoria, Australia

^10^NIHR Oxford Biomedical Research Centre, Oxford University Hospitals NHS Foundation Trust, John Radcliffe Hospital, Oxford, UK

^11^Medical Research Council Population Health Research Unit at the University of Oxford, Nuffield Department of Population Health, University of Oxford, Oxford, UK

^12^Clinical Trial Service Unit & Epidemiological Studies Unit (CTSU), Nuffield Department of Population Health, Big Data Institute Building, Roosevelt Drive, University of Oxford, Oxford, UK

^13^Division of Medicine and Manchester Academic Health Science Centre, Manchester University NHS Foundation Trust Manchester, Manchester, UK

Correspondence: Professor Maciej Tomaszewski, Division of Cardiovascular Sciences, Faculty of Medicine, Biology and Health, University of Manchester, Manchester,

UK E-mail: [maciej.tomaszewski@manchester.ac.uk](mailto:maciej.tomaszewski@manchester.ac.uk)

Phone: 44-161 275 0232

**Table of Contents**

[**Methods** 4](#_Toc83990873)

[**UK Biobank – general characteristics and genotyping** 4](#_Toc83990875)

[**Phenotypes in UK Biobank** 5](#_Toc83990876)

[*Anthropometric measures of obesity* 5](#_Toc83990877)

[*Serum indices of kidney function* 6](#_Toc83990878)

[*Kidney health index* 7](#_Toc83990879)

[**Observational analysis of association between directly measured indices of obesity and kidney phenotypes in UK Biobank** 7](#_Toc83990880)

[**Observational analysis of association between kidney health index and binary traits in UK Biobank** 8](#_Toc83990881)

[**Generation of genetic instruments for the purpose of MR studies** 8](#_Toc83990882)

[**Construction of genetic scores for systolic and diastolic blood pressure in UK Biobank** 10](#_Toc83990883)

[**Genetic scores for type 2 diabetes** 10](#_Toc83990884)

[**Effect of metabolic parameters on kidney function, kidney health index and other kidney diagnoses – one-sample MR in UK Biobank** 10](#_Toc83990885)

[**Effect of obesity on kidney function parameters, CKD and primary and secondary nephropathies – two-sample Mendelian randomisation** 11](#_Toc83990886)

[**Effect of obesity indices on kidney health index – two-sample MR in UK Biobank** 12](#_Toc83990887)

[**Exploration of bidirectional causation** 12](#_Toc83990888)

[**One-sample multivariable Mendelian randomisation analysis** 13](#_Toc83990889)

[**Human kidney tissue collection** 14](#_Toc83990890)

[**Phenotypes** 14](#_Toc83990891)

[**Genotyping and genetic principal components** 15](#_Toc83990892)

[**RNA-sequencing and gene expression data** 15](#_Toc83990893)

[**Analysis of association between expression of kidney gene sets and obesity measures** 16](#_Toc83990894)

[**Analysis of the effect of hypertension and diabetes** 17](#_Toc83990895)

[**Supplementary Figures** 18](#_Toc83990896)

[**FigureS1. Criteria of kidney health index.** 18](#_Toc83990897)

[**FigureS2. Circular representation of information on associations between obesity indices (BMI and WC) and kidney phenotypes.** 20](#_Toc83990898)

[**FigureS3.** **Relationships of BMI (brown) and WC (green) on eGFR (eGFRcrea/eGFRcys/eGFRcreacys) and BUN from observational analyses and one-sample Mendelian randomisation analyses.** 22](#_Toc83990899)

[**FigureS4. The effect of BMI and WC on kidney health index from two-sample Mendelian randomisation analyses.** 24](#_Toc83990900)

[**Acknowledgements** 25](#_Toc83990901)

[**References** 26](#_Toc83990902)

**Methods**

If not specified otherwise, all the statistical analyses were performed using R (version 3.6.2).

**UK Biobank – general characteristics and genotyping**

UK Biobank is a resource with extensive data on 487,395 individuals with a wide range of clinical data linked to genetic information. Detailed information is available on baseline prevalence of many common diseases, demographics, medical and familial history, prenatal exposures, environmental, lifestyle, social and psychological factors, medical therapy, physiological measurements, blood biochemistry and urine analysis as well as a variety of imaging.^1^

In addition, UK Biobank participants underwent a genome-wide profiling – DNA was extracted from blood using a cartridge-based, magnetic bead extraction methodology^2^ and genotyped using either UK Biobank Axiom array or BiLEVE array.^1^ The genotypes underwent standard quality control filters^1^ and were imputed to the Haplotype Reference Consortium (HRC),^3^ Phase 3 1000 Genomes^4^ and UK10K^5^ reference panels by the UKB central analysis group.^1^ At the post-imputation quality control level, we used the quality control criteria and excluded genetic variants if imputation score ≤0·3 or minor allele frequency (MAF) ≤0·01.

Of 487,395 individuals who were genotyped in UK Biobank, we used data for ≈300,000 unrelated white British participants with clinically valid information on the phenotypes of interest to this project – body mass index (BMI), waist circumference (WC), serum creatinine, urinary creatinine, cystatin C, blood urea, urinary albumin and qualitative renal clinical outcomes. We followed UK Biobank sample-based quality control criteria^1^ and excluded samples/individuals if: they were outliers in heterozygosity and missingness, had sample call rate (computed using probesets internal to Affymetrix) <97% or the resolution of the distributions of intensity 'contrast' values <0·82. We also excluded individuals identified as carriers of sex chromosomal abnormalities (configurations other than XX or XY), subjects who had cryptic relatedness with other individuals, or individuals of non-white British genetic ancestry.

For the purpose of accounting for population stratification we used the first 10 genetic principal components generated using 147,604 autosomal single nucleotide variants (SNVs) in 407,219 biologically unrelated individuals of different ethnicities.^1^

**Phenotypes in UK Biobank**

*Anthropometric measures of obesity*

We selected two of the most common anthropometric measures of obesity – BMI and WC. Weight was measured by the Tanita BC-418MA body composition analyser. Height (cm) was measured by a Seca 202 device. BMI was calculated as weight divided by height^2^ (kg/m^2^). WC was measured manually by using flexible plastic tape. We excluded BMI and WC outlier values using modified form of Tukey’s method, (where the upper fence was defined as 3*interquartile range above the third quartile and the lower fence as 3*interquartile range below the first quartile).^6^

*Serum indices of kidney function*

We extracted information on three measures of eGFR in UK Biobank based on: (i) serum creatinine (eGFRcrea), (ii) cystatin C (eGFRcys) and (iii) both creatinine and cystatin C (eGFRcreacys). In brief, creatinine and cystatin C were measured in serum using enzymatic analysis on a Beckman Coulter AU5800 and latex enhanced immunoturbidimetric analysis on a Siemens ADVIA 1800 respectively.^1^ The formulae developed previously were then used to respectively calculate eGFRcrea (141 × min(Scr/κ, 1)^α^ × max(Scr/κ, 1)^−1.209^ × 0·993^Age^ [× 1·018 if female] [× 1·159 if black], where Scr is serum creatinine, κ is 0·7 for females and 0·9 for males, α is −0·329 for females and −0·411 for males, min is the minimum of Scr/κ or 1, and max is the maximum of Scr/κ or 1),^7^ eGFRcys (133 × min(Scys/0·8, 1)^−0.499^ × max (Scys/0·8, 1)^−1.328^ × 0·996^Age^ [× 0·932 if female], where Scys is serum cystatin C, min indicates the minimum of Scr/κ or 1, and max indicates the maximum of Scys/κ or 1)^7^ and eGFRcreacys (135 × min(Scr/κ, 1)^α^ × max(Scr/κ, 1)^−0.601^ × min(Scys/0·8, 1)^−0.375^ × max(Scys/0·8, 1)^−0.711^ × 0·995^Age^ [× 0·963 if female] [× 1·08 if black], where Scr is serum creatinine, Scys is serum cystatin C, κ is 0·7 for females and 0·9 for males, α is −0·248 for females and −0·207 for males, min indicates the minimum of Scr/κ or 1, and max indicates the maximum of Scr/κ or 1).^7^ All eGFR values were winsorised at 15 and 200 ml/min/1·73 m^2^.^8^ We further extracted information on blood urea nitrogen (BUN) (measured by GLDH, kinetic analysis on a Beckman Coulter AU5800) and expressed in mg dl^-1^.^8^ To reduce skewness in the distributions of eGFRcrea, eGFRcys, eGFRcreacys and BUN, the values were log-transformed. Outlier values were then excluded using a modified form of Tukey’s method, (where the upper fence was defined as 1.5*interquartile range above the third quartile and the lower fence as 1.5*interquartile range below the first quartile). The number of individuals with informative values of eGFRcrea, eGFRcys, eGFRcreacys, BUN and was 304,800, 303,373, 317,425 and 314,731, respectively.

*Kidney health index*

We generated a novel composite renal phenotype using information from all available serum measures of kidney function (eGFRcrea, eGFRcys, eGFRcrea, BUN) combined with a urinary bio-marker of kidney damage [albumin-to-creatinine ratio (uACR)] and the International Classification of Disease (ICD)-derived information on the history of kidney disease (Data-field: 41202, 41204) from Hospital Episodes Statistics. Urinary concentrations of albumin and creatinine were measured using immunoturbidimetric analysis and enzymatic analysis respectively on a Beckman Coulter AU5400^1^. The uACR was calculated as urinary albumin (mg/l)/urinary creatinine (mg/dl) × 100 and expressed in mg/g.^9^ Individuals with optimal kidney health were identified if they fulfilled the following criteria: (i) eGFR (eGFRcrea, eGFRcys or eGFRcreacys) ≥60 ml/min/1·73m^2^, (ii) BUN ≤20 mg dl^-1^, (iii) uACR_male_ ≤17 mg/g or uACR_female_ ≤25 mg/g, and (iv) no history of CKD or other kidney diseases (including hypertensive renal disease, renal failure or other disorders of kidney and ureters) based on ICD-derived information from Hospital Episodes Statistics (ICD10 codes: N18, I12, N17-N19 and N25-N29, respectively) (Figure1B). Using these ICD codes, 3,717, 1,268, 7,045 and 2,136 individuals with CKD, hypertensive renal disease, renal failure or other disorders of kidney and ureters were identified in UK Biobank (respectively). A total of 217,289 individuals satisfied the criteria of optimal kidney health index and were defined as having optimal kidney health. The remaining 84,657 individuals did not meet at least one of the above criteria of the kidney health index (and were defined as not having optimal kidney health).

**Observational analysis of association between directly measured indices of obesity and kidney phenotypes in UK Biobank**

To characterise the observational association between directly measured BMI/WC and quantitative serum bio-markers of kidney function (eGFRcrea, eGFRcys, eGFRcreacys, and BUN) we used linear regression. We applied logistic regression to examine the association between BMI/WC and binary kidney phenotypes (i.e. CKD and kidney health index). All regression models were adjusted for age, age^2^, sex, assessment centre and Townsend Deprivation Index.

**Observational analysis of association between kidney health index and binary traits in UK Biobank**

We identified 403 binary traits where the number of cases was larger than 100 using self-reported data and ICD10-derived diagnoses in UK Biobank. These traits were further grouped into 22 clinical categories. To explore the association between directly measured kidney health index (as an independent variable) and each of 403 binary traits (as a dependent variable) in UK Biobank, we applied logistic regression with age, age^2^, sex, assessment centre and Townsend Deprivation Index as covariates. We calculated a correction for multiple testing using the false discovery rate (FDR) – findings with FDR<0·05 were considered statistically significant.

**Generation of genetic instruments for the purpose of MR studies**

We used single nucleotide polymorphisms (SNPs) independently [linkage disequilibrium (LD) R^2^ measure <0·01)] associated with BMI or WC (at P <5×10^−8^) in previous genome-wide association studies conducted in populations of European ancestry other than UK Biobank.^10,11^ After applying these filters and LD-based pruning, 72 and 43 independent SNPs were retained as genetic instruments for BMI and WC, respectively.^12^

We then extracted these genetic instruments from the UK Biobank imputed dataset and calculated genetic scores for BMI and WC by summing up the increasing-trait allele dosages weighted by their relative effect size on adiposity parameters (β_GP_), as reported in their original genome-wide association study (GWAS) (Σ_i_ dosage_GPi_×β_GPi_).^10,11^ All SNPs had an imputation quality score (R^2^) ≥0·9. The SNP effects (β_GP_) used to construct the genetic scores were originally scaled according to a standard deviation increment (SD_X_) of the adiposity trait in the discovery study (SD_BMI_=4·6; SD_wc_=13·3 cm); hence, a one unit increment in the genetic score would indicate one standard deviation increase in the adiposity phenotypes in the discovery sample. The fraction of variance in BMI and WC explained by the SNPs in the discovery study was 2·4% and 1·6%, respectively.^10,11^

To minimise the risk that the causality signal observed from BMI/WC to eGFR may reflect an effect on metabolism of creatinine/cystatin C/BUN, we also re-evaluated associations by excluding SNPs mapping within a distance of 500 Kbp of genes metabolically or mechanistically related to the abundance of these traits. Specifically, ten genes related to creatinine metabolism were identified from the Reactome pathway [“Creatine metabolism” (R-HSA-71288)]. We identified ten genes related to BUN metabolism through the Reactome pathway “Urea cycle” (R-HSA-70635). We also used the Uniprot, Interpro and Ensembl databases to identify all type 2 cystatin genes for exclusion from the causal analysis. As a result of these additional biology-driven investigations, we excluded two SNPs from the BMI instrument and three SNPs from the WC instrument in our further sensitivity analyses testing the effect of BMI/WC on eGFRcys, BUN and kidney health index.

Based on data from previous studies^13,14^ we also used PheLiGe^15^ to determine if any of SNPs selected as BMI/WC instruments show associations with smoking or education in previously conducted GWAS. We identified that 21 and 10 genetic instruments of BMI and 12 and 8 WC genetic instruments were associated (at P<5x10^-4^) with smoking and education phenotypes in PheLiGe, respectively. We then examined the effect of excluding these SNPs from BMI/WC genetic scores on (i) their direct associations with smoking initiation (i.e. comparison of ever-smokers with non-smokers) and cigarettes per day (i.e. number of cigarettes currently smoked daily) as well age of completed full time education in UK Biobank (using linear/logistic regression adjusted for age, age^2^, sex, genotyping array and the first 10 PCs and (ii) the causal associations with kidney health index (see below).The Bonferroni-adjusted P-values were calculated at the experiment level, i.e. (eight analyses on smoking and four on educational attainment).

**Construction of genetic scores for systolic and diastolic blood pressure in UK Biobank**

In the absence of summary statistics for SBP and DBP from GWAS conducted without adjusting for BMI, we applied a block jack-knife weighting approach^16^ to perform GWAS on SBP/DBP in UK Biobank and construct SBP and DBP genetic scores that were not hampered by over-fitting.^16^ First, we generated 10 random subsets of samples within UK Biobank. For each subset, we conducted GWAS within the other 9 subsets. We then built a weighted genetic score for SBP/DBP that was applied to the individual subset excluded from the GWAS. This was repeated until a genetic score was built for all 10 subsets. GWAS of SBP and DBP were conducted using Bolt-LMM,^17^ adjusting for age, age^2^, sex, genotyping array and the first 10 genetic principal components. For measures taken while a subject was on antihypertensive treatment we added 15 mmHg to SBP and 10 mmHg to DBP.^18^ All SNPs selected as genetic instruments for the purpose of building the genetic scores showed an independent (LD R^2^< 0·01) association with the respective phenotype (at P <5×10^−8^ for SBP and DBP) and had an imputation quality score (R^2^)≥0·9. We calculated genetic scores for SBP and DBP by summing up the increasing-trait allele dosages weighted by their relative effect size from the corresponding GWAS.

**Genetic scores for type 2 diabetes**

We used the SNP summary statistics from the most recent and largest (with 1,042,540 individuals of European ancestry) GWAS of type 2 diabetes (T2D)^19^ that was not BMI-adjusted and independent of UK Biobank. All SNPs selected as genetic instruments were independently (LD R2<0·01) associated with T2D (at P<5×10^-8^).

**Effect of metabolic parameters on kidney function, kidney health index and other kidney diagnoses – one-sample MR in UK Biobank**

We first estimated the influence of obesity indices on kidney function measures in a one-sample MR using a two-stage least square approach (2SLS)^20^ with externally-derived genetic scores of BMI and WC as an instrument, measured BMI and WC as an exposure and kidney function parameters (i.e. eGFRcrea, eGFRcys, eGFRcreacys and BUN) as an outcome. The effect of BMI and WC, SBP and DBP, T2D on kidney health index and/or ICD-informed kidney diagnoses (CKD, hypertensive renal disease, renal failure, acute renal failure or other disorders of kidney and ureters, where appropriate) was also tested using the 2SLS approach. Age, age^2^, sex, genotyping array, and first 10 genetic principal components were used as covariates in the above analyses. We calculated a correction for multiple testing at the level of each experiment using FDR – findings with FDR<0·05 were considered statistically significant.

**Effect of obesity on kidney function parameters, CKD and primary and secondary nephropathies – two-sample Mendelian randomisation**

To replicate the estimated causal effects of both obesity indices (as exposures) on biochemical parameters of kidney function we conducted two-sample MR using four different models [inverse variance weighted (IVW) regression, weighted median, RadialMR and MRPRESSO].^21–23^ The weighted median approach is less sensitive to the potentially pleiotropic effects of variants acting as outliers.^21^ RadialMR and MRPRESSO methods detect and remove outlying SNPs from the genetic instruments to account for heterogeneity hence minimise the potential influence of pleiotropic effect. To quantify the magnitude of horizontal pleiotropy we employed MR-Egger intercept test.^21^

For the purpose of this analysis we used GIANT-derived genetic instruments and the respective SNP summary statistics from GWAS of BMI and WC.^10,11^ We extracted independent summary statistics of obesity-associated SNPs from GWAS of log-transformed eGFRcys (n=33,152)^24^ and log-transformed BUN (n=243,031)^8^ conducted by the CKDgen Consortium.^25^ Further investigations on potentially causal effects of obesity on kidney diseases (CKD, IgA nephropathy and diabetic nephropathy) were conducted using the same principles and the same MR models. Independent summary statistics from previous GWAS of these diseases were used to estimate the effect of obesity indices on CKD (n=480,698), IgA nephropathy (n=5,957) and diabetic nephropathy (n=40,340).^8,26,27^ To correct for multiple testing we calculated FDR at the experiment-wide level. Causal effect estimates from at least three of the four MR methods significant after the correction for multiple testing (FDR<0·05) and no evidence of horizontal pleiotropy (FDR>0·05) were set a criterion of evidence for causality. RadialMR and MRPRESSO were performed using R packages RadialMR (version 0.4) and MRPRESSO (version 1.0) respectively. IVW, weighted median and MR-Egger intercept test were implemented in the R package MendelianRandomization (version 0.4.2).

**Effect of obesity indices on kidney health index – two-sample MR in UK Biobank**

As a sensitivity analysis we also conducted two-sample MR using BMI and WC as an exposure and kidney health index as an outcome.

We first conducted GWAS of kidney health index using SAIGE (a generalized mixed model association test). In brief, this test used the saddlepoint approximation to account for case-control imbalance^28^ in the UK Biobank, adjusting for age, age^2^, sex, genotyping array and the first 10 genetic principal components. The summary statistics from our *de novo* GWAS of kidney health index in UK Biobank were then combined with the summary data of genetic instruments for the obesity indices from the independent GWAS^10,11^ in two-sample MR. We used four different MR methods [inverse variance-weighted (IVW) regression, weighted median, RadialMR and MRPRESSO]^21–23^ to estimate the causal effect of BMI and wait circumference on kidney health index. In all these analyses we corrected for multiple testing using FDR and used the criteria of causality as reported above.

**Exploration of bidirectional causation**

We also explored the possibility of reverse causality (i.e. causal effect from kidney health index on obesity indices). We used GWAS summary data for SNP-exposure and SNP-outcome associations (as reported above) to estimate potentially causal effects of eGFRcys, BUN and kidney health index on obesity indices in a set of two-sample MR models. In brief, the most significant independent SNPs (R^2^ <0·01, P <5×10^-8^) associated with kidney health index in UK Biobank were selected for the analyses as instruments. SNP summary statistics from GIANT-GWAS of BMI and WC^10,11^ were used. We corrected for multiple testing using FDR and used the criteria of causality for two-sample MR analyses as reported above.

**One-sample multivariable Mendelian randomisation analysis**

Potentially causal associations of BP and T2D with kidney health index in MR analyses motivated us to quantify the extent to which the effects of obesity indices on kidney health index were independent of BP and T2D. We applied multivariable MR (MVMR) with BMI, WC, SBP, DBP and T2D as exposures and kidney health index as the outcome using a 2SLS approach.^29^ The MVMR estimations measure the direct effect of each exposure on the outcome adjusted for the other exposures included in the model. In our studies we examined several separate combinations of exposures (i) BMI and SBP, (ii) BMI and DBP, (iii) BMI and T2D, (iv) BMI, SBP and T2D (v) BMI, DBP and T2D, (vi) WC and SBP, (vii) WC and DBP, (viii) WC and T2D, (ix) WC, SBP and T2D and (x) WC, DBP and T2D with the genetic scores associated with all exposures included in the derivation of predicted estimates. For example, to conduct a MVMR analysis of the effect of BMI and SBP on kidney health index, we first regressed BMI on the genetic scores for BMI and SBP to obtain a genetically predicted value of BMI that was adjusted for SBP. We repeated this for SBP to derive genetically predicted values of SBP that were adjusted for BMI. We then regressed kidney health index on both genetically predicted BMI and SBP to obtain the direct effect of BMI on kidney health index that was not mediated by SBP. Assuming that obesity has a causal effect on BP^30,31^ we obtained an estimate of the indirect effect of BMI (on the log odds ratio scale) on kidney health index that was mediated by SBP calculated as the difference between the total effect of BMI on kidney health index obtained from the MR estimate and the direct effect of BMI on kidney health index, conditional on BP obtained from the MVMR estimation.^32^ Then the proportion of effect mediated by SBP was derived by dividing the indirect effect by the total effect, with standard error estimated using bootstrapping.^33^ We conducted the MVMR analysis using the same methods to estimate the effect of other combinations of exposures on kidney health index. The strength of the genetic scores to predict the exposures was assessed using the Sanderson–Windmeijer conditional F-statistic.^29^ Age, age^2^, sex, genotyping array, and first 10 genetic principal components were used as covariates. We calculated a correction for multiple testing using FDR – findings with FDR<0·05 were considered statistically significant.

## **Human kidney tissue collection**

A total of 467 human kidney samples with matching clinical information were drawn from kidney tissue resource consisted of five studies [TRANScriptome of renaL humAn TissuE (TRANSLATE) its extension – TRANSLATE-T study, MoleculAr analysis of human kiDney-Manchester renal tIssue pRojEct (ADMIRE), molecular analysis of mechanisms Regulating gene Expression in Post-ischAemic Injury to Renal allograft (REPAIR) and Renal gEne expreSsion and PredispOsition to cardiovascular and kidNey Disease (RESPOND).^34–40^ In brief, TRANSLATE, ADMIRE and RESPOND collected tissue samples from the unaffected part of the kidneys surgically removed due to unilateral renal cancer. TRANSLATE-T and REPAIR collected tissue specimen through a needle biopsy of kidneys prior to the transplantation, as reported before.^35,37,38^ All samples were either immediately immersed in RNAlater (Invitrogen) or snap-frozen after harvesting.

## **Phenotypes**

Demographic and clinical information (age, sex, weight, height, WC, hypertension and diabetes status) were extracted from each study’s database. BMI was calculated by dividing weight (in kg) by height squared (in m^2^). WC was measured using a measuring tape placed around the trunk at the midline level. In TRANSLATE and RESPOND studies, hypertension was defined as BP values ≥140/90 mmHg (measured on at least two separate occasions) and/or being on pharmacological antihypertensive treatment, as reported elsewhere.^36,38^ Diabetes was defined as either self-reported history of diabetes and/or being on hypoglycaemic medications.^34,38^ In ADMIRE, TRANSLATE-T and REPAIR studies, information on history of hypertension and diabetes was based on the available hospital documentation.^38^

## **Genotyping and genetic principal components**

Kidney DNA was extracted using the Qiagen DneasyBlood and Tissue kit following the standard protocol. Genotypes were generated from the Illumina Infinium Human CoreExome BeadChip array. Variant-level quality control of the resultant genotype data was based on the following filtering criteria: missingness > 5%, Hardy-Weinberg equilibrium test P-value <1x10^-3^, minor allele frequency (MAF) < 5% and any position duplicated variants were removed. Sample-level quality control involved removal of all samples with a genotyping rate < 95%, a heterozygosity rate further than 3 standard deviations from the mean, cryptic relatedness to any other sample, inconsistent genetic and phenotypic sex and any non-European genetic ancestry, as reported before.^35^ Genetic principal components (PCs) were calculated using the “—pca” command in PLINK v1.90b with the following filtering criteria for input genotypes: ambiguous genetic variants (C/G or A/T) and MAF > 0.4. We also removed all variants in 24 LD regions and then pruned the final genetic variants using the “—indep-pairwise” method in PLINK with a window size of 10kb, a step size of 50 variants and an r^2^ threshold of 0.05.

## **RNA-sequencing and gene expression data**

Qiagen Rneasy and miRNeasy kits were used to extract RNA from kidney tissue samples, as reported before.^35,38^ Illumina sequencing libraries were generated from 1µg of RNA using a TruSeq poly-A protocol. cDNA libraries were then sequenced on either an Illumina NextSeq or HiSeq 4000 and were found to produce a mean of 32 million paired reads per sample (5.5Gb). Base calling and sequence quality were ascertained and investigated using FastQC (www.bioinformatics.babraham.ac.uk/projects/fastqc). Kallisto^41^ was used to quantify expression at the transcript level, in units of transcripts per million (TPM). Transcript level values were then summed per gene to generate overall gene expression values. Genes were selected for further testing if their expression was greater than 0.1 TPM and had a read count greater than or equal to 6 in 20% of the samples from each of the sample cohorts in our collection. Sequenced samples were excluded that did not have more than 10 million reads or a D-statistic (normalised metric of sample intercorrelation) of greater than 5 or showed ambiguous expression of sex-specific genes. Sample identifiers were also verified by matching genotypes called from the RNA-seq data with array-based genotyping data. These quality control filters retained 22,127 renal genes for further normalisation and analysis. Raw TPM values were log (base 2) transformed (with an offset of 1 added), this was followed by robust quantile normalisation (www.bioconductor.org/packages/release/bioc/html/aroma.light.html) and subsequent standardisation by rank-based inverse normal transformation.^37^

## **Analysis of association between expression of kidney gene sets and obesity measures**

We used multiple linear regression to test 22,127 expressed renal genes for association with BMI and WC as implemented by the “limma” R package (www.bioconductor.org/packages/release/bioc/html/limma.html). The regression model included gene expression as the dependent variable and each of two obesity traits, age, sex, study, 3 genetic principal components, sequencing batch and either 33 or 30 surrogate variables (for BMI and WC, respectively) as independent variables. Surrogate variables (to control for unmeasured and unwanted sources of variation in gene expression) were derived by the “sva” R package^42^ using the gene expression data and the regression model used by limma, the number of surrogate variables was automatically determined by sva. This approach reduces potential cofounding effects resulting from technical artefacts or latent biological variables (including those of environmental, genetic or clinical origin).^38,42^ We then applied gene set enrichment analysis, as implemented by the “fgsea” R package (www.bioconductor.org/packages/release/bioc/html/fgsea.html), using the “canonical pathways” gene set v7.2 from MsigDB (www.gsea-msigdb.org/gsea/msigdb/index.jsp) and 100,000 permutations for significance testing. Input to fgsea was the full set of HGNC gene symbols and their associated t-statistics from the limma regression model. Significantly enriched pathways were defined as those with a false discovery rate (FDR) adjusted P-value less than 0.05. Normalised enrichment scores (NES) were calculated by fgsea and indicate directionality of gene expression change in a pathway. Leading edge genes were also determined by fgsea as the genes that contributed to a statistically significant enrichment statistic for a specific pathway. Results from the two obesity traits: WC and BMI were combined, and we retained the full set of pathway associations from both traits. Information on biological roles of pathways, together with the similarity in identity of defining leading edge genes, were then used to classify each pathway into overarching biological themes, in line with our previously reported strategy.^43^

## **Analysis of the effect of hypertension and diabetes**

To investigate the effect of hypertension and diabetes on renal gene expression pathways we re-ran the GSEA of obesity traits with an addition of hypertension or diabetes status as an additional covariate. The significantly associated pathways from the unadjusted analysis were then compared to the results from the hypertension and diabetes-adjusted analyses. The replication of association results was determined by matching on the exact pathway identifier from MsigDB, by having the same direction of normalised enrichment score (NES) and retaining the statistical significance with the obesity measure after adjustment for multiple testing. Any pathways retaining their directionally consistent FDR-adjusted association with BMI or WC after inclusion of hypertension or diabetes in the models were deemed as independent to both hypertension and diabetes (respectively) while those whose association was no longer statistically significant after the adjustment were interpreted as being mediated specifically either by hypertension or diabetes.

**Supplementary Figures**

**
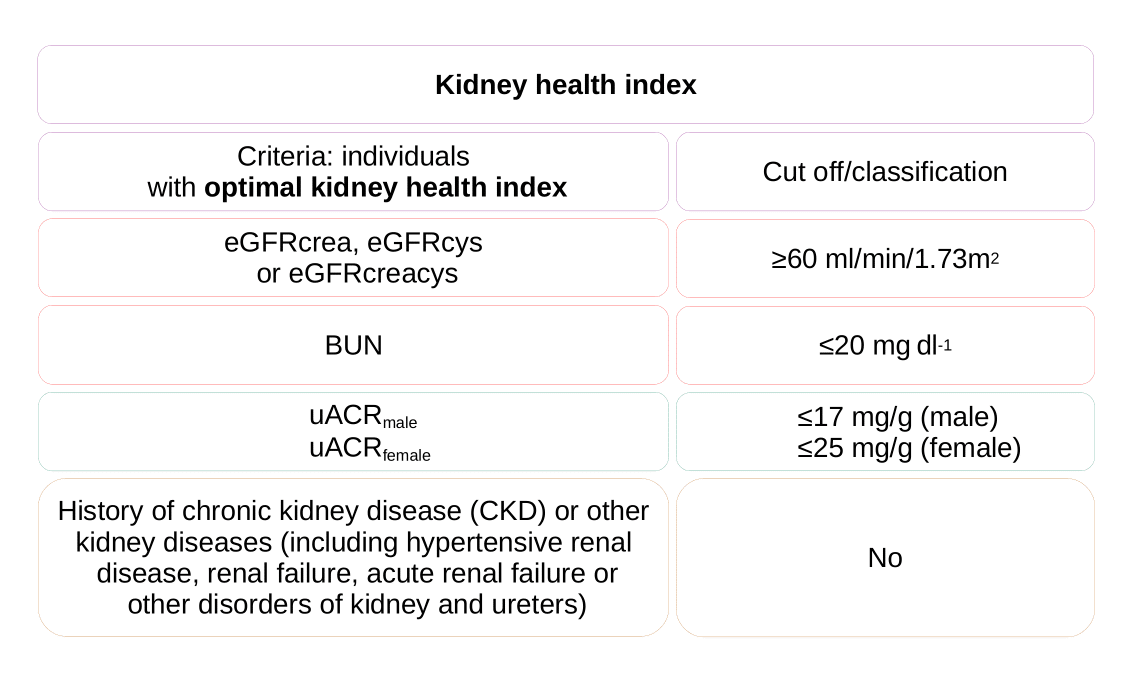
**

**FigureS1. Criteria of kidney health index.** eGFRcrea – GFR estimated by creatinine, eGFRcys – GFR estimated by cystatin C, eGFRcreacys – GFR estimated by creatinine and cystatin C, BUN – blood urea nitrogen.

**
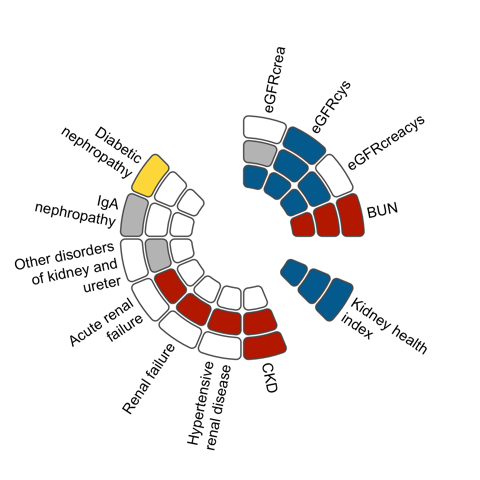
**

**FigureS2. Circular representation of information on associations between obesity indices (BMI and WC) and kidney phenotypes.** From outermost to innermost data circle we show: associations from two-sample MR, one-sample MR and observational analysis respectively. Associations were coloured as: white – not tested, grey – not associated with obesity indices, yellow – only associated with BMI, red – positively associated with both obesity indices, blue – negatively associated with obesity indices. eGFRcrea – GFR estimated by creatinine, eGFRcys – GFR estimated by cystatin C, eGFRcreacys – GFR estimated by creatinine and cystatin C, BUN – blood urea nitrogen, CKD – chronic kidney disease.


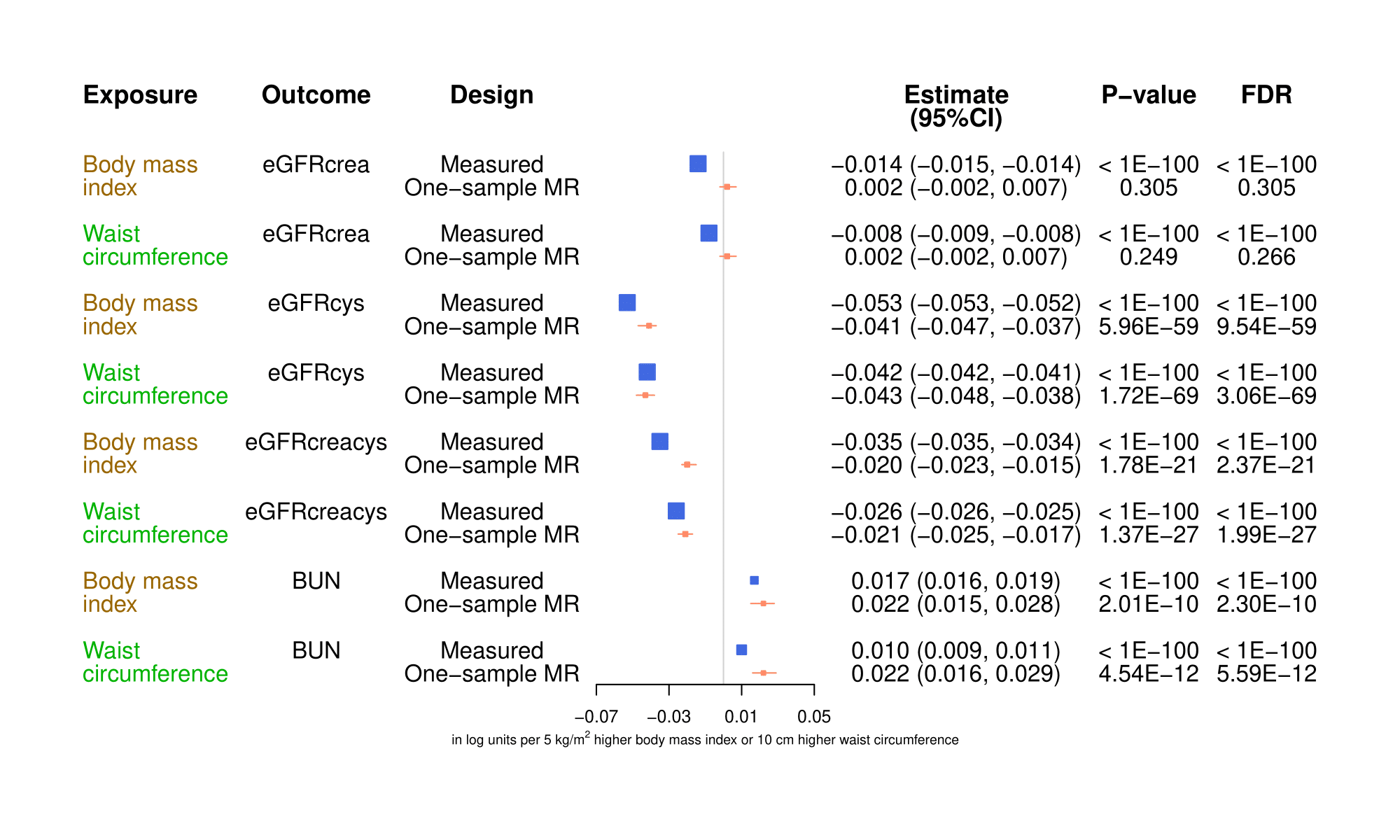


**FigureS3.** **Relationships of BMI (brown) and WC (green) on eGFR (eGFRcrea/eGFRcys/eGFRcreacys) and BUN from observational analyses and one-sample Mendelian randomisation analyses.** Two different methods were coloured as: blue – observational analysis, orange – one-sample MR. MR – Mendelian randomisation, Estimate – effect size of each body weight measure on eGFR and BUN (in log units per 5 kg/m^2^ higher BMI or 10 cm higher WC), CI – confidence interval, P-value – level of statistical significance, FDR – false discovery rate.

**
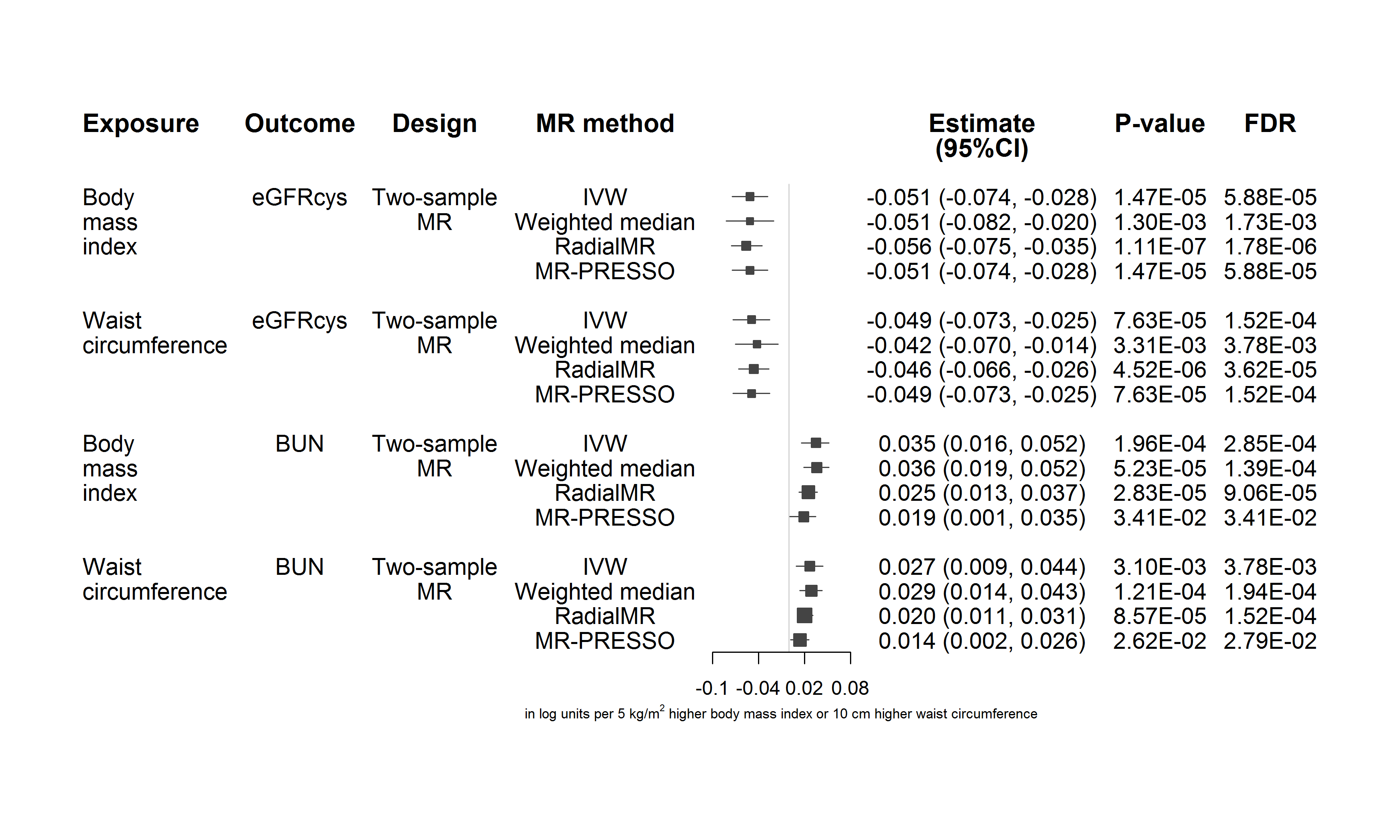
**

**FigureS4. The effect of BMI and WC on kidney health index from two-sample Mendelian randomisation analyses.** eGFRcys – eGFR calculated by cystatine C, BUN – blood urea nitrogen, MR – Mendelian randomisation, IVW – inverse variance weighted, Pleiotropy – horizontal pleiotropy tested using MR-Egger intercept, β – regression coefficient (in log units per 5 kg/m^2^ higher BMI or 10 cm higher WC), 95%CIL – 95% confidence interval lower bound, 95%CIU – 95% confidence interval upper bound, P-value – level of statistical significance, FDR – false discovery rate.

**Acknowledgements**

Human Kidney Tissue Resource:

The following investigators contributed to recruitment and/or phenotyping of human kidney gene expression studies: Wojciech Wystrychowski^1^, Monika Szulinska^2^, Andrzej Antczak^3^, Maciej Glyda^4^, Robert Król^1^, Joanna Zywiec^5^, Ewa Zukowska-Szczechowska^6^, Pawel Bogdanski^2,^ Bernard Keavney^7^

1 Department of General, Vascular and Transplant Surgery, Medical University of Silesia, Katowice, Poland

2 Department of Treatment of Obesity, Metabolic Disorders and Clinical Dietetics, Poznan University of Medical Sciences, Poznan, Poland.

3 Department of Urology and Uro-oncology, Karol Marcinkowski University of Medical Sciences, Poznan, Poland

4 Department of Transplantology and General Surgery Poznan, Collegium Medicum, Nicolaus Copernicus University, Bydgoszcz, Poland

5 Department of Internal Medicine, Diabetology and Nephrology, Medical University of Silesia, Zabrze, Poland

6 Department of Health Care, Silesian Medical College, Katowice, Poland

7^1^Division of Cardiovascular Sciences, Faculty of Medicine, Biology and Health, University of Manchester, Manchester, UK

**References**

1. Bycroft C, Freeman C, Petkova D, Band G, Elliott LT, Sharp K, Motyer A, Vukcevic D, Delaneau O, O’Connell J, Cortes A, Welsh S, Young A, Effingham M, McVean G, Leslie S, Allen N, Donnelly P, Marchini J. The UK Biobank resource with deep phenotyping and genomic data. *Nature* 2018;**562**:203–209.

2. Welsh S, Peakman T, Sheard S, Almond R. Comparison of DNA quantification methodology used in the DNA extraction protocol for the UK Biobank cohort. *BMC Genomics* 2017;**18**:26.

3. McCarthy S, Das S, Kretzschmar W, Delaneau O, Wood AR, Teumer A, Kang HM, Fuchsberger C, Danecek P, Sharp K, Luo Y, Sidore C, Kwong A, Timpson N, Koskinen S, Vrieze S, Scott LJ, Zhang H, Mahajan A, Veldink J, Peters U, Pato C, Duijn CM van, Gillies CE, Gandin I, Mezzavilla M, Gilly A, Cocca M, Traglia M, Angius A, et al. A reference panel of 64,976 haplotypes for genotype imputation. *Nat Genet* 2016;**48**:1279–1283.

4. 1000 Genomes Project Consortium, Auton A, Brooks LD, Durbin RM, Garrison EP, Kang HM, Korbel JO, Marchini JL, McCarthy S, McVean GA, Abecasis GR. A global reference for human genetic variation. *Nature* 2015;**526**:68–74.

5. Walter K, Min JL, Huang J, Crooks L, Memari Y, McCarthy S, Perry JRB, Xu C, Futema M, Lawson D, Iotchkova V, Schiffels S, Hendricks AE, Danecek P, Li R, Floyd J, Wain LV, Barroso I, Humphries SE, Hurles ME, Zeggini E, Barrett JC, Plagnol V, Brent Richards J, Greenwood CMT, Timpson NJ, Durbin R, Soranzo N, Bala S, Clapham P, et al. The UK10K project identifies rare variants in health and disease. *Nature* 2015;**526**:82–90.

6. Beyer H. Tukey, John W.: Exploratory Data Analysis. Addison-Wesley Publishing Company Reading, Mass. — Menlo Park, Cal., London, Amsterdam, Don Mills, Ontario, Sydney 1977, XVI, 688 S. *Biom J* 1981;**23**:413–414.

7. Inker LA, Schmid CH, Tighiouart H, Eckfeldt JH, Feldman HI, Greene T, Kusek JW, Manzi J, Van Lente F, Zhang YL, Coresh J, Levey AS. Estimating Glomerular Filtration Rate from Serum Creatinine and Cystatin C. *N Engl J Med* 2012;**367**:20–29.

8. Wuttke M, Li Y, Li M, Sieber KB, Feitosa MF, Gorski M, Tin A, Wang L, Chu AY, Hoppmann A, Kirsten H, Giri A, Chai J-F, Sveinbjornsson G, Tayo BO, Nutile T, Fuchsberger C, Marten J, Cocca M, Ghasemi S, Xu Y, Horn K, Noce D, Most PJ van der, Sedaghat S, Yu Z, Akiyama M, Afaq S, Ahluwalia TS, Almgren P, et al. A catalog of genetic loci associated with kidney function from analyses of a million individuals. *Nat Genet* 2019;**51**:957–972.

9. Teumer A, Li Y, Ghasemi S, Prins BP, Wuttke M, Hermle T, Giri A, Sieber KB, Qiu C, Kirsten H, Tin A, Chu AY, Bansal N, Feitosa MF, Wang L, Chai J-F, Cocca M, Fuchsberger C, Gorski M, Hoppmann A, Horn K, Li M, Marten J, Noce D, Nutile T, Sedaghat S, Sveinbjornsson G, Tayo BO, Most PJ van der, Xu Y, et al. Genome-wide association meta-analyses and fine-mapping elucidate pathways influencing albuminuria. *Nat Commun* 2019;**10**:4130.

10. Locke AE, Kahali B, Berndt SI, Justice AE, Pers TH, Day FR, Powell C, Vedantam S, Buchkovich ML, Yang J, Croteau-Chonka DC, Esko T, Fall T, Ferreira T, Gustafsson S, Kutalik Z, Luan J, Mägi R, Randall JC, Winkler TW, Wood AR, Workalemahu T, Faul JD, Smith JA, Zhao JH, Zhao W, Chen J, Fehrmann R, Hedman ÅK, Karjalainen J, et al. Genetic studies of body mass index yield new insights for obesity biology. *Nature* 2015;**518**:197–206.

11. Shungin D, Winkler TW, Croteau-Chonka DC, Ferreira T, Locke AE, Mägi R, Strawbridge RJ, Pers TH, Fischer K, Justice AE, Workalemahu T, Wu JMW, Buchkovich ML, Heard-Costa NL, Roman TS, Drong AW, Song C, Gustafsson S, Day FR, Esko T, Fall T, Kutalik Z, Luan J, Randall JC, Scherag A, Vedantam S, Wood AR, Chen J, Fehrmann R, Karjalainen J, et al. New genetic loci link adipose and insulin biology to body fat distribution. *Nature* 2015;**518**:187–196.

12. Carreras-Torres R, Johansson M, Haycock PC, Relton CL, Smith GD, Brennan P, Martin RM. Role of obesity in smoking behaviour: Mendelian randomisation study in UK Biobank. *BMJ* 2018;**361**:k1767.

13. Censin JC, Peters SAE, Bovijn J, Ferreira T, Pulit SL, Mägi R, Mahajan A, Holmes MV, Lindgren CM. Causal relationships between obesity and the leading causes of death in women and men. *PLOS Genet* 2019;**15**:e1008405.

14. Zhu P, Herrington WG, Haynes R, Emberson J, Landray MJ, Sudlow CLM, Woodward M, Baigent C, Lewington S, Staplin N. Conventional and Genetic Evidence on the Association between Adiposity and CKD. *J Am Soc Nephrol* 2021;**32**:127–137.

15. Shashkova TI, Pakhomov ED, Gorev DD, Karssen LC, Joshi PK, Aulchenko YS. PheLiGe: an interactive database of billions of human genotype–phenotype associations. *Nucleic Acids Res* 2020;**49**:D1347–D1350.

16. Shao J, Wu CFJ. A General Theory for Jackknife Variance Estimation. *Ann Stat* 1989;**17**:1176–1197.

17. Loh P-R, Tucker G, Bulik-Sullivan BK, Vilhjálmsson BJ, Finucane HK, Salem RM, Chasman DI, Ridker PM, Neale BM, Berger B, Patterson N, Price AL. Efficient Bayesian mixed-model analysis increases association power in large cohorts. *Nat Genet* 2015;**47**:284–290.

18. Newton-Cheh C, Johnson T, Gateva V, Tobin MD, Bochud M, Coin L, Najjar SS, Zhao JH, Heath SC, Eyheramendy S, Papadakis K, Voight BF, Scott LJ, Zhang F, Farrall M, Tanaka T, Wallace C, Chambers JC, Khaw K-T, Nilsson P, Harst P van der, Polidoro S, Grobbee DE, Onland-Moret NC, Bots ML, Wain LV, Elliott KS, Teumer A, Luan J, Lucas G, et al. Eight blood pressure loci identified by genome-wide association study of 34,433 people of European ancestry. *Nat Genet* 2009;**41**:666–676.

19. Vujkovic M, Keaton JM, Lynch JA, Miller DR, Zhou J, Tcheandjieu C, Huffman JE, Assimes TL, Lorenz K, Zhu X, Hilliard AT, Judy RL, Huang J, Lee KM, Klarin D, Pyarajan S, Danesh J, Melander O, Rasheed A, Mallick NH, Hameed S, Qureshi IH, Afzal MN, Malik U, Jalal A, Abbas S, Sheng X, Gao L, Kaestner KH, Susztak K, et al. Discovery of 318 new risk loci for type 2 diabetes and related vascular outcomes among 1.4 million participants in a multi-ancestry meta-analysis. *Nat Genet* 2020;**52**:680–691.

20. Burgess S, Small DS, Thompson SG. A review of instrumental variable estimators for Mendelian randomization. *Stat Methods Med Res* 2017;**26**:2333–2355.

21. Burgess S, Bowden J, Dudbridge F, Thompson SG. Robust instrumental variable methods using multiple candidate instruments with application to Mendelian randomization. *ArXiv160603729 Stat* 2016;

22. Bowden J, Spiller W, Del Greco M F, Sheehan N, Thompson J, Minelli C, Davey Smith G. Improving the visualization, interpretation and analysis of two-sample summary data Mendelian randomization via the Radial plot and Radial regression. *Int J Epidemiol* 2018;**47**:1264–1278.

23. Verbanck M, Chen C-Y, Neale B, Do R. Detection of widespread horizontal pleiotropy in causal relationships inferred from Mendelian randomization between complex traits and diseases. *Nat Genet* 2018;**50**:693–698.

24. Pattaro C, Teumer A, Gorski M, Chu AY, Li M, Mijatovic V, Garnaas M, Tin A, Sorice R, Li Y, Taliun D, Olden M, Foster M, Yang Q, Chen M-H, Pers TH, Johnson AD, Ko Y-A, Fuchsberger C, Tayo B, Nalls M, Feitosa MF, Isaacs A, Dehghan A, Adamo P d’, Adeyemo A, Dieffenbach AK, Zonderman AB, Nolte IM, Most PJ van der, et al. Genetic associations at 53 loci highlight cell types and biological pathways relevant for kidney function. *Nat Commun* 2016;**7**:10023.

25. Köttgen A, Pattaro C. The CKDGen Consortium: ten years of insights into the genetic basis of kidney function. *Kidney Int* 2020;**97**:236–242.

26. Feehally J, Farrall M, Boland A, Gale DP, Gut I, Heath S, Kumar A, Peden JF, Maxwell PH, Morris DL, Padmanabhan S, Vyse TJ, Zawadzka A, Rees AJ, Lathrop M, Ratcliffe PJ. HLA Has Strongest Association with IgA Nephropathy in Genome-Wide Analysis. *J Am Soc Nephrol* 2010;**21**:1791–1797.

27. Zuydam NR van, Ahlqvist E, Sandholm N, Deshmukh H, Rayner NW, Abdalla M, Ladenvall C, Ziemek D, Fauman E, Robertson NR, McKeigue PM, Valo E, Forsblom C, Harjutsalo V, Perna A, Rurali E, Marcovecchio ML, Igo RP, Salem RM, Perico N, Lajer M, Käräjämäki A, Imamura M, Kubo M, Takahashi A, Sim X, Liu J, Dam RM van, Jiang G, Tam CHT, et al. A Genome-Wide Association Study of Diabetic Kidney Disease in Subjects With Type 2 Diabetes. *Diabetes* 2018;**67**:1414–1427.

28. Zhou W, Nielsen JB, Fritsche LG, Dey R, Gabrielsen ME, Wolford BN, LeFaive J, VandeHaar P, Gagliano SA, Gifford A, Bastarache LA, Wei W-Q, Denny JC, Lin M, Hveem K, Kang HM, Abecasis GR, Willer CJ, Lee S. Efficiently controlling for case-control imbalance and sample relatedness in large-scale genetic association studies. *Nat Genet* 2018;**50**:1335–1341.

29. Sanderson E, Davey Smith G, Windmeijer F, Bowden J. An examination of multivariable Mendelian randomization in the single-sample and two-sample summary data settings. *Int J Epidemiol* 2019;**48**:713–727.

30. Holmes MV, Lange LA, Palmer T, Lanktree MB, North KE, Almoguera B, Buxbaum S, Chandrupatla HR, Elbers CC, Guo Y, Hoogeveen RC, Li J, Li YR, Swerdlow DI, Cushman M, Price TS, Curtis SP, Fornage M, Hakonarson H, Patel SR, Redline S, Siscovick DS, Tsai MY, Wilson JG, van der Schouw YT, FitzGerald GA, Hingorani AD, Casas JP, de Bakker PIW, Rich SS, et al. Causal Effects of Body Mass Index on Cardiometabolic Traits and Events: A Mendelian Randomization Analysis. *Am J Hum Genet* 2014;**94**:198–208.

31. Lyall DM, Celis-Morales C, Ward J, Iliodromiti S, Anderson JJ, Gill JMR, Smith DJ, Ntuk UE, Mackay DF, Holmes MV, Sattar N, Pell JP. Association of Body Mass Index With Cardiometabolic Disease in the UK Biobank: A Mendelian Randomization Study. *JAMA Cardiol* 2017;**2**:882–889.

32. Carter AR, Sanderson E, Hammerton G, Richmond RC, Smith GD, Heron J, Taylor AE, Davies NM, Howe LD. Mendelian randomisation for mediation analysis: current methods and challenges for implementation. *bioRxiv* 2020;835819.

33. Bowden J, Davey Smith G, Haycock PC, Burgess S. Consistent Estimation in Mendelian Randomization with Some Invalid Instruments Using a Weighted Median Estimator. *Genet Epidemiol* 2016;**40**:304–314.

34. Rowland J, Akbarov A, Eales J, Xu X, Dormer JP, Guo H, Denniff M, Jiang X, Ranjzad P, Nazgiewicz A, Prestes PR, Antczak A, Szulinska M, Wise IA, Zukowska-Szczechowska E, Bogdanski P, Woolf AS, Samani NJ, Charchar FJ, Tomaszewski M. Uncovering genetic mechanisms of kidney aging through transcriptomics, genomics, and epigenomics. *Kidney Int* 2019;**95**:624–635.

35. Eales JM, Jiang X, Xu X, Saluja S, Akbarov A, Cano-Gamez E, McNulty MT, Finan C, Guo H, Wystrychowski W, Szulinska M, Thomas HB, Pramanik S, Chopade S, Prestes PR, Wise1 I, Evangelou E, Salehi M, Shakanti Y, Ekholm M, Denniff M, Nazgiewicz A, Eichinger F, Godfrey B, Antczak A, Glyda M, Kró R, Eyre S, Brown J, Berzuini C, Bowes J, Caulfield M, Zukowska-Szczechowska E, Zywiec J, Bogdanski P, Kretzler M, Woolf AS, Talavera D, Keavney B, Maffia P, Guzik TJ, O’Keefe RT, Trynka G, Samani NJ, Hingorani A, Sampson MG, Morris AP, Charchar FJ and Tomaszewski M. Uncovering genetic mechanisms of hypertension through multi-omic analysis of the kidney. *Nat Genet* 2021;https://doi.org/10.1038/s41588-021-00835-w.

36. Tomaszewski M, Eales J, Denniff M, Myers S, Chew GS, Nelson CP, Christofidou P, Desai A, Büsst C, Wojnar L, Musialik K, Jozwiak J, Debiec R, Dominiczak AF, Navis G, Gilst WH van, Harst P van der, Samani NJ, Harrap S, Bogdanski P, Zukowska-Szczechowska E, Charchar FJ. Renal Mechanisms of Association between Fibroblast Growth Factor 1 and Blood Pressure. *J Am Soc Nephrol* 2015;**26**:3151–3160.

37. Xu X, Eales JM, Akbarov A, Guo H, Becker L, Talavera D, Ashraf F, Nawaz J, Pramanik S, Bowes J, Jiang X, Dormer J, Denniff M, Antczak A, Szulinska M, Wise I, Prestes PR, Glyda M, Bogdanski P, Zukowska-Szczechowska E, Berzuini C, Woolf AS, Samani NJ, Charchar FJ, Tomaszewski M. Molecular insights into genome-wide association studies of chronic kidney disease-defining traits. *Nat Commun* 2018;**9**:1–12.

38. Jiang X, Eales JM, Scannali D, Nazgiewicz A, Prestes P, Maier M, Denniff M, Xu X, Saluja S, Cano-Gamez E, Wystrychowski W, Szulinska M, Antczak A, Byars S, Skrypnik D, Glyda M, Król R, Zywiec J, Zukowska-Szczechowska E, Burrell LM, Woolf AS, Greenstein A, Bogdanski P, Keavney B, Morris AP, Heagerty A, Williams B, Harrap SB, Trynka G, Samani NJ, et al. Hypertension and renin-angiotensin system blockers are not associated with expression of angiotensin-converting enzyme 2 (ACE2) in the kidney. *Eur Heart J* 2020;**41**:4580–4588.

39. Morris AP, Le TH, Wu H, Akbarov A, Most PJ van der, Hemani G, Smith GD, Mahajan A, Gaulton KJ, Nadkarni GN, Valladares-Salgado A, Wacher-Rodarte N, Mychaleckyj JC, Dueker ND, Guo X, Hai Y, Haessler J, Kamatani Y, Stilp AM, Zhu G, Cook JP, Ärnlöv J, Blanton SH, Borst MH de, Bottinger EP, Buchanan TA, Cechova S, Charchar FJ, Chu P-L, Damman J, et al. Trans-ethnic kidney function association study reveals putative causal genes and effects on kidney-specific disease aetiologies. *Nat Commun* 2019;**10**:29.

40. Marques FZ, Romaine SP, Denniff M, Eales J, Dormer J, Garrelds IM, Wojnar L, Musialik K, Duda-Raszewska B, Kiszka B, Duda M, Morris BJ, Samani NJ, Danser AJ, Bogdanski P, Zukowska-Szczechowska E, Charchar FJ, Tomaszewski M. Signatures of miR-181a on the Renal Transcriptome and Blood Pressure. *Mol Med* 2015;**21**:739–748.

41. Bray NL, Pimentel H, Melsted P, Pachter L. Near-optimal probabilistic RNA-seq quantification. *Nat Biotechnol* 2016;**34**:525–527.

42. Leek JT, Johnson WE, Parker HS, Jaffe AE, Storey JD. The sva package for removing batch effects and other unwanted variation in high-throughput experiments. *Bioinforma* 2012;**28**:882–883.

43. Eales JM, Maan Akhlaq A., Xu X, Michoel T, Hallast P, Batini C, Zadik D, Prestes PR, Molina E, Denniff M, Schroeder J, Bjorkegren JLM, Thompson J, Maffia P, Guzik TJ, Keavney Bernard, Jobling MA., Samani NJ, Charchar FJ, Tomaszewski M. Human Y Chromosome Exerts Pleiotropic Effects on Susceptibility to Atherosclerosis. *Arterioscler Thromb Vasc Biol* 2019;**39**:2386–2401.
